# Toward Developing a Standardized Core Set of Outcome Measures in Mobile Health Interventions for Tuberculosis Management: Systematic Review

**DOI:** 10.2196/12385

**Published:** 2019-02-19

**Authors:** Seohyun Lee, Youngji Lee, Sangmi Lee, Sheikh Mohammed Shariful Islam, Sun-Young Kim

**Affiliations:** 1 Institute of Health & Environment, Center for Global Health Research Graduate School of Public Health Seoul National University Seoul Republic of Korea; 2 Department of Public Health Sciences Graduate School of Public Health Seoul National University Seoul Republic of Korea; 3 Institute for Physical Activity and Nutrition School of Exercise and Nutrition Sciences, Faculty of Health Deakin University Melbourne Australia

**Keywords:** mHealth, tuberculosis, outcome measures, evidence synthesis, low-and middle-income countries

## Abstract

**Background:**

Tuberculosis (TB) management can be challenging in low- and middle-income countries (LMICs) not only because of its high burden but also the prolonged treatment period involving multiple drugs. With rapid development in mobile technology, mobile health (mHealth) interventions or using a mobile device for TB management has gained popularity. Despite the potential usefulness of mHealth interventions for TB, few studies have quantitatively synthesized evidence on its effectiveness, presumably because of variability in outcome measures reported in the literature.

**Objective:**

The aim of this systematic review was to evaluate the outcome measures reported in TB mHealth literature in LMICs.

**Methods:**

MEDLINE, EMBASE, and the Cochrane Database of Systematic Reviews were searched to identify mHealth intervention studies for TB (published up to May 2018) that reported any type of outcome measures. The extracted information included the study setting, types of mHealth technology used, target population, study design, and categories of outcome measures. Outcomes were classified into 13 categories including treatment outcome, adherence, process measure, perception, technical outcome, and so on. The qualitative synthesis of evidence focused on the categories of outcome measures reported by the type of mHealth interventions.

**Results:**

A total of 27 studies were included for the qualitative synthesis of evidence. The study designs varied widely, ranging from randomized controlled trials to economic evaluations. A total of 12 studies adopted short message service (SMS), whereas 5 studies used SMS in combination with additional technologies or mobile apps. The study populations were also diverse, including patients with TB, patients with TB/HIV, health care workers, and general patients attending a clinic. There was a wide range of variations in the definition of outcome measures across the studies. Among the diverse categories of outcome measures, treatment outcomes have been reported in 14 studies, but only 6 of them measured the outcome according to the standard TB treatment definitions by the World Health Organization.

**Conclusions:**

This critical evaluation of outcomes reported in mHealth studies for TB management suggests that substantial variability exists in reporting outcome measures. To overcome the challenges in evidence synthesis for mHealth interventions, this study can provide insights into the development of a core set of outcome measures by intervention type and study design.

## Introduction

Tuberculosis (TB) is one of the deadly infectious diseases that have claimed millions of lives worldwide. According to the World Health Organization (WHO), globally, there were 10.4 million new TB cases causing approximately 1.2 million deaths in 2016 [1,2]. The mortality rate of TB is disproportionately higher in low- and middle-income countries (LMICs). Over 95% of TB deaths occurred in these countries, and 7 LMICs (India, Indonesia, China, Philippines, Pakistan, Nigeria, and South Africa) accounted for 64% of the total burden [3]. In fact, previous studies have shown that there is empirical evidence of positive associations between poverty indicators and TB incidence both at the macro and individual levels [4]. Considering the vicious cycle of poverty and TB, alleviating the burden of TB is more challenging for the LMICs because it requires adequate resources for “prolonged treatment with multiple drugs [5].” The 6-month course of first-line therapy can be burdensome with the possibility of adverse reactions and the treatment of multidrug-resistant (MDR)-TB requires more toxic and expensive drugs [6]. For example, the cost of bedaquiline, a second-line medication to treat MDR-TB, was US $3000 per treatment in middle-income countries and US $900 in low-income countries [7]. In fact, premature discontinuation of the treatment, which can lead to MDR-TB, is common among TB patients not only for its toxicity but also for socioeconomic costs associated with it [8]. Therefore, the management of TB is notoriously difficult especially in LMICs.

In this context, using mobile devices for TB treatment has been recognized as an innovative approach for LMICs where mobile subscription rates have dramatically increased over the past decade. Mobile health (mHealth) interventions involving mobile devices in the management of TB have the potential for reducing costs of information delivery and improving the quality of communication [9]. mHealth can be useful for TB treatment adherence support such as short message service (SMS) for medication reminders or mobile apps for remote directly observed treatment (DOT) strategy [10,11].

Despite the potential of mHealth interventions for improving TB management, the empirical evidence on its effectiveness is mixed. Some studies have demonstrated the effectiveness and feasibility of the mHealth interventions for TB [12,13], whereas others have shown no significant impact [14,15]. Moreover, no study has attempted to synthesize the results quantitatively to rigorously evaluate the effectiveness of mHealth in TB management. Presumably, one of the reasons for such difficulty in synthesizing and evaluating the findings comes from wide variations in the outcomes reported from the mHealth studies for TB.

To respond to this knowledge gap, this study aimed to systematically review previous mHealth studies for TB management and critically evaluate and categorize the outcome measures for different mobile technologies and study designs. The goal of this study was to provide researchers insights into the development of a core set of outcome measures for mHealth interventions intended to improve TB treatment adherence. In doing so, the study can facilitate the evidence synthesis of mHealth interventions for TB.

## Methods

### Search Strategy and Review Process

Electronic databases (MEDLINE, EMBASE, and Cochrane Database of Systematic Reviews) were searched to identify peer-reviewed studies of mHealth interventions for TB. The systematic search was supplemented by reviewing relevant review papers identified from the initial search. The search strategy for the study population includes key terms describing LMICs such as “resource poor” or “developing country.” The search strategy for the mHealth intervention combined multiple keywords such as “mHealth” and “text-messaging.” As for the target disease, “tuberculosis,” “TB,” “multi-drug resistant tuberculosis,” and “MDR-TB” were used as search terms. No restrictions were applied to the publication type or publication date, but the language filter was applied to identify studies published in English. The search included articles published up to May 2018. The full search strategy is available in Multimedia Appendix 1.

Furthermore, 3 authors (ShL, YL, and SmL) independently reviewed the retrieved studies throughout the selection process. Each study identified from the databases was screened by 2 reviewers and then a full-text review was conducted for the potentially eligible studies. The disagreement on the selection process was resolved by the other authors who were not involved in the review of the specific study under discussion.

### Eligibility for Review

The inclusion criteria for this systematic review were as follows: First, studies conducted in the context of LMICs, as defined by the World Bank’s income cutoffs [16]; second, studies involving an intervention using mobile devices (ie, mHealth intervention); third, the target disease of the study should be TB or MDR-TB; fourth, studies designed to evaluate the effectiveness or benefits of mHealth interventions for TB (eg, observational study, mixed-methods study or implementation project, randomized controlled trial [RCT]); fifth, studies reporting more than one type of outcome; finally, only full-text studies published in English were considered eligible. In addition, the authors attempted to identify individual studies from reviews or systematic reviews, which were included in this study.

### Data Extraction and Analysis

The qualitative synthesis of evidence focused on the outcome measures reported in each type of mHealth intervention. Information about the study setting, mHealth technologies used, target populations, and types of outcome measures was extracted. To classify the diverse types of detailed outcome measures, the following categories were used: (1) treatment outcome; (2) treatment outcome as defined by WHO; (3) adherence; (4) process measure; (5) perception; (6) technical outcome; (7) health outcome; (8) quality of life; (9) knowledge; (10) cost-effectiveness; (11) cost; (12) psychosocial outcome; and (13) mortality. Some explanations about these measures are provided in the next few paragraphs.

The treatment outcome includes any outcome measure that deals with the result of TB treatment, such as sputum smear conversion or microscopy test result. The treatment outcome following the WHO definition was separately categorized [17]. The WHO definition was developed to make a distinction for treatment outcomes between the drug-susceptible TB and drug-resistant TB, which are mutually exclusive groups. According to the WHO definition, any patient with TB should belong to either group and then 1 of the 7 treatment outcome cohorts: (1) cured; (2) treatment completed; (3) treatment failed; (4) died; (5) lost to follow-up; (6) not evaluated; and (7) treatment success. The WHO definitions for each of these 7 categories differ between the drug-susceptible TB and drug-resistant TB as described in Multimedia Appendix 2.

The adherence outcome includes medication adherence or treatment adherence. The process measure is any outcome measure related to treatment process, including the receipt of diagnostic test, attendance to appointments, or reporting of adverse events. The perception indicates any outcome measure that captures the user’s thoughts on mHealth for TB management. The technical outcome relates to the outcome measures that investigate the technical feasibility such as processing times or system installation. Health outcome, quality of life, knowledge (eg, patients’ understanding of the disease or the technology), cost-effectiveness (ie, the extent to which an alternative provides value for money), cost (ie, costs associated with an intervention from different perspectives), psychosocial outcome, and mortality outcomes are additional categories which are self-explanatory.

### Risk of Bias: Quality Assessment

To evaluate the quality of individual studies included for our review, risk of bias was assessed with the existing tools. As this systematic review includes various types of studies, it is important to have a coherent set of quality assessment tools for different study designs. Therefore, we used the modified version of the Critical Appraisal Skills Program (CASP) that provides the checklists specific to various types of studies ranging from RCTs and qualitative studies to economic evaluation [18]. In case of mixed-methods studies whose CASP tool has not been developed yet, the quality assessment criteria for mixed-methods studies from the previous study were employed [19]. The quality assessment of studies is presented in Multimedia Appendix 3.

## Results

### Overview of Included Studies

**Figure 1:**
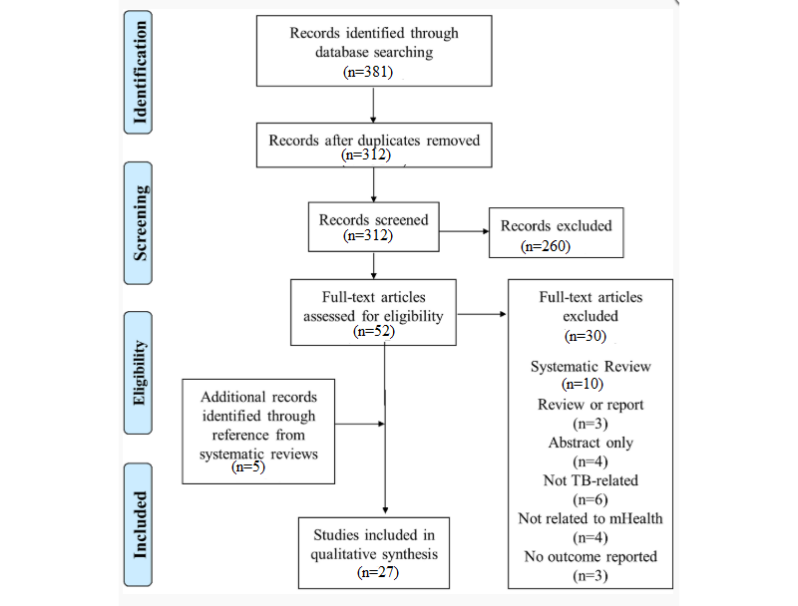
Flow diagram for selection process following the Preferred Reporting Items for Systematic Reviews and Meta-Analyses (PRISMA) guideline. mHealth: mobile health; TB: tuberculosis.

Among the 312 studies identified after removing duplicates, 260 articles were excluded during the screening process based on the titles and abstracts. Therefore, 52 articles were assessed for eligibility through a full-text review. Of those, 27 studies were included for the qualitative synthesis of evidence. A flow diagram for the selection process, based on the Preferred Reporting Items for Systematic Reviews and Meta-Analyses guidelines, is provided in Figure 1 [20].

### Qualitative Synthesis of Evidence

Table 1 presents the results of qualitative synthesis of the mHealth studies for TB management. Approximately, half (15 out of 27) of the studies were conducted in African countries. The study designs were diverse, including 6 RCTs, 5 mixed-methods studies, 1 cohort study, 3 qualitative studies, 4 observational studies, 6 implementation projects, and 2 economic evaluation studies. The types of mHealth technologies utilized were diverse as well: 12 studies employed SMS, 6 studies used mobile app, 5 studies used SMS plus other technology, 3 studies utilized phone calls, and only 1 study applied mHealth for mobile data collection. With regard to the study population, a majority (20 out of 27) of the studies were targeted for TB patients or TB/HIV patients, but there were a few studies that examined the experience of health care workers or general patients at the clinic for TB test results notification.

In terms of the outcome measures, there was a wide range of variants in their definitions even within each category of the outcomes. For instance, both Mohammed et al and Bediang et al defined treatment success as the primary outcome measure of SMS intervention for TB medication adherence in their RCT, but they defined treatment success differently [15,22]. Mohammed et al defined it as “the sum of patients clinically reported as cured (ie, a patient whose sputum smear or culture was positive at the beginning of treatment but who was smear- or culture-negative in the last month of treatment and on at least one previous occasion) or treatment completed (ie, a patient who completed treatment but who does not have a negative sputum smear or culture result in the last month of treatment and on at least one previous occasion)” [22]. On the contrary, Bediang et al defined treatment success as “having completed 6 months treatment and having negative sputum smears at 5 months” [15].

### Summary of Outcome Measures

Table 2 summarizes the types of outcome measures reported in the included studies, using the categories defined in this review. The most frequently reported outcome type was treatment success. Approximately half of the studies reviewed (14 out of 27) included treatment outcome but only 6 studies among them followed the WHO definition for the treatment outcome (Multimedia Appendix 2). The second most frequently reported category was perception on the mHealth intervention (13 out of 27). Other categories reported were diverse and included technical outcome, medication or treatment adherence, process measure, etc. However, there was substantial variability within each category of outcome, as shown in Table 1. For example, acceptability and satisfaction within the perception category were defined differently from one study to another [15,28-30,32,35,36,40,44]. Also, SMS-only intervention studies did not focus on technical outcome [12,15,22,29,30,32,35, 36,42,43,45,46] whereas studies involving other mHealth technologies such as an app or mobile data collection did so [21,28,33,37,39-41]. On the contrary, outcomes related to cost or cost-effectiveness were reported only via studies involving SMS [29,45,46].

**Table 1:** Summary of included studies.

Source	Country	Study design	Mobile health technology	Population	Outcome measures	Outcome category	Purpose
Blaya, 2009 [21]	Peru	RCT^a^	Mobile data collection	Health centers	Processing times, frequency of errors, the number of work-hours expended by data collectors	Technical outcome	For laboratory data collection
Mohammed, 2016 [22]	Pakistan	RCT	SMS^b^	TB^c^ patients	Primary: clinically recorded treatment success based upon intention-to-treat; Secondary: treatment outcomes (WHO definitions^d^), self-reported medication adherence, self-reported psychological and physical health measures	Treatment outcome, adherence, health outcome	For medication adherence
Bassett, 2013 [23]; Bassett, 2016 [24]	South Africa	RCT protocol (2013); RCT (2016)	SMS and phone calls	Patients at clinic	Primary: treatment completion; Secondary: mortality, receipt of CD4 count and TB test results, and repeat CD4 counts for those not antiretroviral therapy (ART)–eligible at baseline	Treatment outcome, mortality, process measure	For appointment and test result reminder and psychosocial support
Huang, 2017 [25]	China	Cluster RCT protocol	Mobile app	TB patients	Primary: TB treatment result (WHO definitions^d^); Secondary: treatment adherence (the percentage of patients receiving TB treatment who missed fewer than 5% of doses), self-reported adherence, knowledge about TB, quality of life (QoL)	Treatment outcome, adherence, knowledge, QoL	For Bracelet- and self-directed observational therapy
Bediang, 2014 [26]; Bediang, 2018 [15]	Cameroon	RCT protocol (2014); RCT (2018)	SMS	TB patients	Primary: cure rate (absence of Koch’s bacilli in the sputum), treatment success (having completed 6 months' treatment and having negative sputum smears at 5 months); Secondary: treatment adherence (drug prescriptions collected and doses taken), attendance to appointments, punctuality of appointments, treatment outcome (WHO definitions^d^), the number of participants who develop resistance, satisfaction	Treatment outcome, adherence, process measure, perception	For medication adherence
Khachadourian, 2015 [27]	Armenia	RCT protocol	SMS and phone calls	TB patients	Primary: physician-reported treatment outcome (WHO definitions^d^); Secondary: patients’ knowledge, depression, QoL, within-family TB-related stigma, family social support, self-reported treatment adherence	Treatment outcome, knowledge, psychosocial outcome, QoL, adherence	For medication adherence
Chaiyachati, 2013 [28]	South Africa	Mixed-methods study	Mobile app	Health care workers	Primary: proportion of weekly adverse events forms submitted vs expected by mobile health care workers; Secondary: acceptability (perceived comfort levels with using mobile phone technology), quality of adverse events monitoring, proportion of reportable adverse events being captured; Technical outcomes: phone usage patterns, technical problems experienced	Process measure, perception, technical outcome	For adverse events reporting
Howard, 2016 [29]	Lesotho	Mixed-methods, cluster-randomized trial protocol	SMS	TB/HIV patients, health care workers	Primary: ART initiation, retention, and TB treatment success; Secondary: time to ART initiation, adherence, change in cluster of differentiation 4 (CD4) count, sputum smear conversion, cost-effectiveness, acceptability	Process measure, treatment outcome, adherence, cost-effectiveness, perception	For treatment adherence
Iribarren, 2013 [30]	Argentina	Mixed-methods study (including RCT)	SMS	TB patients	Primary: feasibility (access to mobile phones, familiarity with texting, rate of participant refusal, suboptimal TB understanding), and acceptability (feeling cared for patient’s treatment, self-reporting adherence); Secondary: initial efficacy (microscopy test result from positive to negative, treatment outcome)	Process measure, perception, adherence, treatment outcome	For treatment adherence
Hirsch-Moverman, 2017a [31]	Lesotho, Ethiopia	Mixed-methods; implementation science study	Phone calls	TB/HIV patients, TB patients	The number of call attempts per participant for each month, completeness of monthly calls, success rates, challenges	Process measure	For medication adherence
Hirsch-Moverman, 2017b [32]	Lesotho	Mixed-methods implementation science study, cluster-randomized trial protocol	SMS	TB patients, health care workers, caregivers	Primary: the number of child contacts Isoniazid preventive therapy (IPT) initiation, IPT completion; Secondary: HIV testing, yield of active prevalent TB among child contacts, acceptability, and utilization of community-based intervention components	Treatment outcome, perception, process measure	For medication adherence and appointment reminders
Nguyen, 2017 [33]	Vietnam	Cohort study	SMS and mobile app	TB patients	Primary: proportion of patients completing all doses of self-administered treatment; Secondary: proportion of videos uploaded as scheduled, proportion of patients discontinuing using Video DOT (VDOT)	Treatment outcome, technical outcome, process measure	For medication adherence
Daftary, 2017 [34]	Ethiopia	Qualitative study	Interactive voice response (IVR)	HIV patients	Perceptions and attitude, perceived benefits and challenges	Perception	For preventive therapy adherence
Albino, 2014 [35]	Peru	Qualitative study	SMS	TB patients	Perceptions and acceptability	Perception	For treatment adherence
Nhavoto, 2017 [36]	Mozambique	Qualitative study	SMS	TB patients, Health care workers	Usefulness, perceived benefits, ease of use, satisfaction, risks of the SMS system	Perception	For treatment adherence
Hoffman, 2010 [37]	Kenya	Observational study	Mobile app	TB patients	Primary: technical feasibility (patient and health provider receptivity to remote directly observed treatment [DOT]); Secondary: patient preferences and receptivity to receiving TB health message on a mobile phone	Technical outcome, perception	For Mobile Direct Observation of Treatment
de Sumari-de Boer, 2016 [12]	Tanzania	Observational pilot study	SMS	HIV patients, TB patients	Quantitative: percentage of doses taken on time, percentage of sent reminders (divided by total intake prescription), percentage of correct reminders (after missed doses), percentage of incorrect reminders (after opening the pillbox but the signal was not sent), percentage of extra openings, percentage of missed doses, percentage of adherence with the exclusion of doses that were taken after a reminder; Qualitative: general experience with using the device	Process measure, adherence, perception	For medication adherence
Garfein, 2015 [38]	Mexico, USA	Observational pilot study	Mobile app	TB patients	Primary: adherence rate (the number of medication doses observed in videos divided by the number of doses expected during the treatment period); Secondary: perceptions of VDOT	Adherence, perception	For VDOT
Dwolatzky, 2006 [39]	South Africa	Observational pilot study	Mobile app	Patients at clinic	Time taken to locate the households	Technical outcome	For locating patients’ homes by global positioning system and personal digital assistant
Ha, 2016 [40]	Botswana	Implementation project	Mobile app	TB patients	Cases screened for contact tracing, time required to complete TB contact tracing per contact, quality of data collected, user satisfaction with usability, operational considerations	Technical outcome, perception	For contact tracing
Cowan, 2016 [41]	Mozambique	Implementation project	SMS and mobile app	Health facility	System installation on computers, development of Web-based interface and automated SMS and email messages, test results uploaded to the system, SMS notifications sent to key personnel, the number of users	Technical outcome	For remote monitoring solution
Kunawararak, 2011 [14]	Thailand	Implementation project	Phone calls	TB patients	Cure rates, completion rates, failure rates and success rates, conversion rates	Treatment outcome	For medication adherence
Lorent, 2014 [42]	Cambodia	Implementation project	SMS	General population	TB case detection- smear-positivity, clinical TB treatment uptake–time to treatment initiation outcome–treatment outcomes (WHO definitions^d^), delay in linkage to care	Process measure, treatment outcome	For test result notification
Mahmud, 2010 [43]	Malawi	Implementation project	SMS	Health care workers	Operational net savings, worker time gained, patient enrollment	Process measure	For continuity of care
Narasimhan, 2014 [44]	India	Implementation project	SMS and phone calls	TB patients	Treatment completion and cure rates (WHO definitions^d^), treatment adherence rates, adverse drug reaction rates, stigma associated with TB, patient satisfaction, usage of the mHealth initiative	Treatment outcome, adherence, perception	For medication adherence
Broomhead, 2012 [45]	South Africa	Cost minimization analysis	SMS	TB patients	Smear conversion rate, TB cure rate, reduced average cost per patient	Treatment outcome, cost	For treatment adherence
Hunchangsith, 2012 [46]	Thailand	Cost-effective-ness analysis	SMS	TB patients	Disability-adjusted life years (DALYs) averted, costs (health care perspective), effects of interventions, success rate, failure rate, transfer out rate, death rate	Health outcome, treatment outcome, cost, cost-effective-ness	For medication adherence

^a^RCT: randomized controlled trial.

^b^SMS: short message service.

^c^TB: tuberculosis.

^d^World Health Organization definitions: presented in Multimedia Appendix 2.

**Table 2:** Reported outcomes by mHealth intervention type.

Intervention type (number of studies) and reference		Categories of outcome measure												
		Treatment outcome	Treatment outcome by WHO^a^ definition	Adherence	Process measure	Perception	Technical outcome	Health outcome	QoL^b^	Knowledge	Cost-effectiveness	Cost	Psychosocial outcome	Mortality
**Short message service (SMS; 12 studies)**														
	[22]	✓^c^	✓	✓	—^d^	—	—	✓	—	—	—	—	—	—
	[15]	✓	✓	✓	✓	✓	—	—	—	—	—	—	—	—
	[29]	✓	—	✓	✓	✓	—	—	—	—	✓	—	—	—
	[30]	✓	—	✓	✓	✓	—	—	—	—	—	—	—	—
	[32]	✓	—	—	✓	✓	—	—	—	—	—	—	—	—
	[35]	—	—	—	—	✓	—	—	—	—	—	—	—	—
	[36]	—	—	—	—	✓	—	—	—	—	—	—	—	—
	[12]	—	—	✓	✓	✓	—	—	—	—	—	—	—	—
	[42]	✓	✓	—	✓	—	—	—	—	—	—	—	—	—
	[43]	—	—	—	✓	—	—	—	—	—	—	—	—	—
	[45]	✓	—	—	—	—	—	—	—	—	—	✓	—	—
	[46]	✓	—	—	—	—	—	✓	—	—	✓	✓	—	—
**SMS plus others (5 studies)**														
	[24]	✓	—	—	✓	—	—	—	—	—	—	—	—	✓
	[27]	✓	✓	✓	—	—	—	—	✓	✓	—	—	✓	—
	[33]	✓	—	—	✓	—	✓	—	—	—	—	—	—	—
	[41]	—	—	—	—	—	✓	—	—	—	—	—	—	—
	[44]	✓	✓	✓	—	✓	—	—	—	—	—	—	—	—
**Mobile app (6 studies)**														
	[25]	✓	✓	✓	—	—	—	—	✓	✓	—	—	—	—
	[28]	—	—	—	✓	✓	✓	—	—	—	—	—	—	—
	[37]	—	—	—	—	✓	✓	—	—	—	—	—	—	—
	[38]	—	—	✓	—	✓	—	—	—	—	—	—	—	—
	[39]	—	—	—	—	—	✓	—	—	—	—	—	—	—
	[40]	—	—	—	—	✓	✓	—	—	—	—	—	—	—
**Phone calls or interactive voice response (3 studies)**														
	[31]	—	—	—	✓	—	—	—	—	—	—	—	—	—
	[34]	—	—	—	—	✓	—	—	—	—	—	—	—	—
	[14]	✓	—	—	—	—	—	—	—	—	—	—	—	—
**Mobile data collection (1 study)**														
	[21]	—	—	—	—	—	✓	—	—	—	—	—	—	—

^a^WHO: World Health Organization.

^b^QoL: quality of life.

^c^Tick marks indicate that the specific category of outcome measure was reported.

^d^Outcome measure was not reported.

## Discussion

### Principal Findings

This systematic review critically evaluated the outcomes reported in mHealth studies for TB management in LMICs. The reason why rigorous evidence synthesis is warranted is that recent literature for TB reports mixed results despite the rapid implementation of mHealth technology for TB management. The fragmented pieces of evidence on the effectiveness partly resulted from the wide variations in the definitions of outcome measures in TB mHealth interventions. Even though treatment outcome has been reported by many studies, they often did not adopt the standard definition recommended by the WHO [17].

The WHO definition of TB treatment outcome is part of an effort to standardize outcome measures for TB at the global level. To promote the use of standardized sets of outcome measures for TB, WHO provided the standard definitions and classifications of TB in terms of diagnosis or treatment outcomes [17]. The intention for this standardization effort was to coordinate international comparison of TB treatment outcomes through health information systems. However, the findings of our review revealed that mHealth studies for TB have not comprehensively adopted this standardized approach for TB treatment. Only 6 out of 27 interventions chose to report the treatment outcome according to the WHO definition. Interventions involving phone calls, interactive voice response, or mobile data collection did not consider the WHO definition.

Our findings also suggest that, to rigorously evaluate the effectiveness of mHealth interventions for TB, future studies should be carefully designed with regard to the selection of outcome measures. Indeed, using standard definitions for outcome measures within some commonly reported categories can improve comparability across different studies. As assessed in this study, examples of such categories include treatment outcome (preferably using the WHO definition), perception, process measure, adherence, and technical outcome.

The value of this systematic review can be found in its potential to motivate and facilitate consensus on standard definitions of outcome measures used in mHealth interventions for TB so that such effort can guide more effective mHealth intervention designs for improving TB management. Although current literature shows considerable variability in the definition of outcome measures, discussion and coordination among researchers can promote standardized methods in measuring outcomes. Specifically, the outcomes should be comparable, promote transparent communication, and maintain consistency in terminology. Coordination at the global level is necessary to develop a core set of outcome measures for TB mHealth interventions by study design and technology type utilized.

When choosing a core set of outcome measures with standard definitions, there are additional issues to consider. First, the time point for reporting outcome measures should be clinically meaningful and feasible [47]. Second, a detailed description of the measure should be provided, such as calculation method or definitions. Third, a clear explanation on the target population for each outcome measure can be useful. For example, some outcome measures may be more appropriate for MDR-TB patients rather than TB patients on their first-line therapy course. Finally, long-term outcome measures should be considered to establish fundamental evidence for TB mHealth interventions. The long-term outcomes can be related to physical, psychosocial, or mental health. Despite its importance, our review showed that only 2 out of 27 studies reported long-term health outcomes; Mohammed et al, reported self-reported psychological and physical health measures [22] and Hunchangsith et al, reported DALYs averted [46].

Another issue to consider is related to evidence for cost. As this study suggested, insufficient evidence exists in terms of cost-effectiveness or cost of mHealth interventions for TB management. Those previous studies that have evaluated the cost-effectiveness or cost of mHealth interventions only considered SMS as mHealth channels and did not consider or evaluate other mHealth channels and technologies. However, other mHealth channels and technologies such as mobile apps or global positioning system are now available for TB patients [48]. Therefore, future studies need to assess the cost-effectiveness of such channels and technologies for improving TB management in LMICs.

This study has some limitations. First, the database used for identifying relevant studies is limited to the 3 most frequently cited sources, namely MEDLINE, EMBASE, and Cochrane Database of Systematic Reviews. Grey literature or other sources of information can supplement our findings. To complement this limitation, we attempted to identify additional related studies from relevant systematic reviews searched from our study. Second, the effectiveness of the mHealth interventions for TB was not quantitatively evaluated because of the heterogeneity of the outcomes reported.

Despite these limitations, this study provides an overview of the currently reported outcome measures for mHealth interventions intended to improve TB management in the context of LMICs. The results from this review can be used as a starting point for discussion to adopt standardized definitions within different categories of outcome measures for future mHealth interventions for TB management in LMICs.

### Conclusions

This systematic review of mHealth studies for TB suggests that substantial variability exists with regard to the definitions of outcome measures across studies. Our review highlights that a standardized method for measuring the different outcomes is warranted to improve comparability of outcome measures across studies for a more rigorous and reliable evaluation of the effectiveness of mHealth interventions for TB. In doing so, the coordination among researchers and the development of a core set of outcome measures based on standardized methods would be necessary. Our study provides useful information for researchers to better assess the effectiveness of mHealth interventions for TB. In addition, the study provides insights into the possibility of developing a core set of outcome measures by intervention type and study design based on a standardized or coordinated set of methods.

## Appendix

Multimedia Appendix 1 Search strategy.

Multimedia Appendix 2 Definitions of TB treatment outcome by the WHO [17].

Multimedia Appendix 3 Risk of bias assessment.

## Abbreviations

DALY: disability-adjusted life years
DOT: directly observed treatment
IPT: isoniazid preventive therapy
IVR: interactive voice response
LMICs: low- and middle-income countries
MDR-TB: multidrug resistant-TB
mHealth: mobile health
RCT: randomized controlled trial
SMS: short message service
TB: tuberculosis
WHO: World Health Organization
