# Supplementary material for: Toward Developing a Standardized Core Set of Outcome Measures in Mobile Health Interventions for Tuberculosis Management: Systematic Review
Source: JMIR Mhealth Uhealth. 2019 Feb 19;7(2):e12385. doi: 10.2196/12385 (PMC6399569; doi:10.2196/12385)
Supplement: Multimedia Appendix 1 [file mhealth_v7i2e12385_app1.pdf]

Appendix A. Search Strategy

Pubmed (MEDLINE)			
1	**Population**	"developing countries"[MeSH Terms] OR "developing countries"[All Fields] OR "developing country"[All Fields]	114598
2		lmic OR "low and middle income" OR "low income" OR "lower middle income" OR "middle income" OR "upper middle income"	39589
3		"resource poor" OR "poor resource" OR "resource limited" OR "resource constrained" OR "low resource"	16085
4		#1 OR #2 OR #3	**159148**
5	**Intervention**	"mobile health"[tiab] OR "mhealth"[tiab] OR "m-health"[tiab]	4001
6		"mobile phone" OR "cell phone" OR "cellular phone" OR "smart phone"	10878
7		"mobile device" OR "wearable device" OR "tablet" OR "laptop" OR "ipad" OR "iphone"	26328
8		"SMS" OR "short message service" OR "MMS" OR "multimedia message service"	10599
9		"text messaging" OR "text message" OR "instant message" OR "voice message" OR "phone call" OR "e-mail"[tiab] OR "email"[tiab]	14178
10		("mobile app" OR "mobile apps" OR "mobile application" OR "mobile applications" OR "smartphone app" OR "smartphone apps" OR "smartphone application" OR "smartphone applications")	5579
11		#5 OR #6 OR #7 OR #8 OR #9 OR #10	**64152**
12	**Target disease**	(TB OR tuberculosis OR MDRTB OR Multi-drug-resistant tuberculosis)	**259012**
13		#4 AND #11 AND #12	**71**

EMBASE			
1	**Population**	"developing countries"/exp OR "developing countries" OR "developing country"	125,812
2		lmic OR "low and middle income" OR "low income" OR "lower middle income" OR "middle income" OR "upper middle income"	46,479
3		"resource poor" OR "poor resource" OR "resource limited" OR "resource constrained" OR "low resource"	20,841
4		#1 OR #2 OR #3	**183,660**
5	**Intervention**	"mobile health":ab,ti OR "mhealth":ab,ti OR "m-health":ab,ti	2,990
6		"mobile phone" OR "cell phone" OR "cellular phone" OR "smart phone"	16,587
7		"mobile device" OR "wearable device" OR "tablet" OR "laptop" OR "ipad" OR "iphone"	76,802
8		"SMS" OR "short message service" OR "MMS" OR "multimedia message service"	16,452
9		"text messaging" OR "text message" OR "instant message" OR "voice message" OR "phone call" OR "e-mail":ab,ti OR "email":ab,ti	26,866
10		("mobile app" OR "mobile apps" OR "mobile application" OR "mobile applications" OR "smartphone app" OR "smartphone apps" OR "smartphone application" OR "smartphone applications")	7,881
11		#5 OR #6 OR #7 OR #8 OR #9 OR #10	**137,529**
12	**Target disease**	(TB OR tuberculosis OR MDRTB OR Multi-drug-resistant tuberculosis)	**303,582**
13		#4 AND #11 AND #12	**100**

COCHRANE			
1	**Population**	MeSH descriptor: [Developing Countries] explode all trees<br>developing countries or developing country (Word variations have been searched)<br>#1 or #2	11602
2		lmic OR "low and middle income" OR "low income" OR "lower middle income" OR "middle income" OR "upper middle income"	4862
3		"resource poor" OR "poor resource" OR "resource limited" OR "resource constrained" OR "low resource"	1783
4		#1 OR #2 OR #3	**16016**
5	**Intervention**	mobile health:ti,ab,kw or mhealth:ti,ab,kw or m-health:ti,ab,kw (Word variations have been searched)	1870
6		"mobile phone" OR "cell phone" OR "cellular phone" OR "smart phone"	1686
7		"mobile device" or "wearable device" or "tablet" or "laptop" or "ipad" or "iphone"	13179
8		"SMS" OR "short message service" OR "MMS" OR "multimedia message service"	1581
9		"text messaging" or "text message" or "instant message" or "voice message" or "phone call" or "email":ti,ab,kw or "e-mail":ti,ab,kw (Word variations have been searched)	4987
10		"mobile app" OR "mobile apps" OR "mobile application" OR "mobile applications" OR "smartphone app" OR "smartphone apps" OR "smartphone application" OR "smartphone applications"	935
11		#5 OR #6 OR #7 OR #8 OR #9 OR #10	**20857**
12	**Target disease**	"TB" OR "tuberculosis" or "MDRTB" or "Multi-drug-resistant tuberculosis"	**8950**
13		#4 AND #11 AND #12	**209**
