# Supplementary material for: Toward Developing a Standardized Core Set of Outcome Measures in Mobile Health Interventions for Tuberculosis Management: Systematic Review
Source: JMIR Mhealth Uhealth. 2019 Feb 19;7(2):e12385. doi: 10.2196/12385 (PMC6399569; doi:10.2196/12385)
Supplement: Multimedia Appendix 2 [file mhealth_v7i2e12385_app2.pdf]

Appendix B. Definitions of TB treatment outcome by the WHO [17]

Treatment outcomes for TB patients (except those treated for RR-TB[a] or MDR-TB)

Outcome	Definition
Cured	A pulmonary TB patient with bacteriologically confirmed TB at the beginning of treatment who was smear- or culture-negative in the last month of treatment and on at least one previous occasion.
Treatment completed	A TB patient who completed treatment without evidence of failure but with no record to show that sputum smear or culture results in the last month of treatment and on at least one previous occasion were negative either because tests were not done or because results are unavailable.
Treatment failed	A TB patient whose sputum smear or culture is positive at month 5 or later during treatment.
Died	A TB patient who dies for any reason before starting or during the course of treatment.
Lost to follow-up	A TB patient who did not start treatment or whose treatment was interrupted for 2 consecutive months or more.
Not-evaluated	A TB patient for whom no treatment outcome is assigned. This includes cases “transferred out” to another treatment unit as well as cases for whom the treatment outcome is unknown to the reporting unit.
Treatment success	The sum of cured and treatment completed.
[a]RR-TB: rifampicin-resistant TB	

Treatment outcomes for RR-TB/MDR-TB/XDR-TB[b] patients treated using second-line treatment

Outcome	Definition
Cured	Treatment completed as recommended by the national policy without evidence of failure and three or more consecutive cultures taken at least 30 days apart are negative after the intensive phase.
Treatment completed	Treatment completed as recommended by the national policy without evidence of failure but no record that three or more consecutive cultures taken at least 30 days apart are negative after the intensive phase.
Treatment failed	Treatment terminated or need for permanent regimen change of at least two anti-TB drugs because of:<br>- Lack of conversion by the end of the intensive phase, or<br>- Bacteriological reversion in the continuation phase after conversion to negative, or<br>- Evidence of additional acquired resistance to fluoroquinolones or second-line injectable drugs, or<br>- Adverse drug reactions (ADRs)
Died	A patient who dies for any reason during the course of treatment.
Lost to follow-up	A patient whose treatment was interrupted for 2 consecutive months or more.
Not evaluated	A patient for whom no treatment outcome is assigned. (This includes cases “transferred out” to another treatment unit and whose treatment outcome is unknown)
Treatment success	The sum of *cured* and *treatment completed*.
[b]XDR-TB: extensively drug-resistant TB	
