# Supplementary material for: Toward Developing a Standardized Core Set of Outcome Measures in Mobile Health Interventions for Tuberculosis Management: Systematic Review
Source: JMIR Mhealth Uhealth. 2019 Feb 19;7(2):e12385. doi: 10.2196/12385 (PMC6399569; doi:10.2196/12385)
Supplement: Multimedia Appendix 3 [file mhealth_v7i2e12385_app3.pdf]

Appendix C. Risk of Bias Assessment

Risk of bias assessment for randomized controlled trial (RCT)
CASP (Randomized Controlled Trial) Checklist

**Reference**	Blaya JA, Cohen T, Rodríguez P, Kim J, Fraser HSF. Personal digital assistants to collect tuberculosis bacteriology data in Peru reduce delays, errors, and workload, and are acceptable to users: cluster randomized controlled trial. International journal of infectious diseases : IJID : official publication of the International Society for Infectious Diseases. 2009 12/18;13(3):410-8. PMID: PMC2673336. doi: 10.1016/j.ijid.2008.09.015.

Section A: Are the results of the trial valid?

Question	Comments	Answer
**1. Did the trial address a clearly focused issue?**	The aim of the study was clearly stated: "To evaluate the effectiveness of a personal digital assistant (PDA)-based system for collecting tuberculosis test results and to compare this new system to the previous paper-based system."	**Yes** ☑<br>**Can't tell** ☐<br>**No** ☐
**2. Was the assignment of patients to treatments randomized?**	The study was designed as a cluster randomized controlled trial: "After collecting baseline data for 19 months from four of five health districts in Lima, Peru, we randomly assigned two to the intervention, while two were maintained as controls."	**Yes** ☑<br>**Can't tell** ☐<br>**No** ☐
**3. Were all of the patients who entered the trial properly accounted for at its conclusion?**	All patient-level data were collected according to the study period: "During the intervention period, we collected data on the same endpoints in both control and intervention arms. For the between-districts comparison, we collected all culture and their respective smear microscopy results for the 6 months after the full implementation of the PDA-based system (result dates between March 24 and September 24, 2006)."	**Yes** ☑<br>**Can't tell** ☐<br>**No** ☐
**4. Were patients, health workers and study personnel 'blind' to treatment?**	No information available.	**Yes** ☐<br>**Can't tell** ☑<br>**No** ☐
**5. Were the groups similar at the start of the trial?**	The districts were compared for any potential difference that can affect processing times: "We therefore analyzed the between-districts data and found that there was no statistically significant difference between the mean collection times in the intervention and the control districts. This allowed us to conclude that the time taken for the team to visit the health establishment and collect the result did not contribute to the difference in	**Yes** ☑<br>**Can't tell** ☐<br>**No** ☐

	processing times seen in the between-districts comparison."<br>"The number of years working in the bacteriology team (mean 4.5 vs. 4.9 years) and years of internet experience (mean 4.3 vs. 4.6 years) were similar before and after the PDA-based system was implemented, primarily because three team members participated in all periods of the study."	
**6. Aside from the experimental intervention, were the groups treated equally?**	Information on intervention and control design was provided (PDA vs. paper-based system) but it is difficult to tell if the two groups were treated equally or not.	**Yes** ☐<br>**Can't tell** ☑<br>**No** ☐

Section B: What are the results?

Question	Comments
**7. How large was the treatment effect?**	The effect size was relatively large: "The PDA-based system had a significant effect on processing times ($p < 0.001$) and errors ($p = 0.005$). In the between-districts comparison, the median processing time for cultures was reduced from 23 to 8 days and for smears was reduced from 25 to 12 days. In that comparison, the proportion of cultures with delays >90 days was reduced from 9.2% to 0.1% and the number of errors was decreased by 57.1%. The intervention reduced the work-hours necessary to process results by 70% and was preferred by all users."
**8. How precise was the estimate of the treatment effect?**	No information available.

Section C: Will the results help locally?

Question	Comments	Answer
**9. Can the results be applied to the local population, or in your context?**	The feasibility of PDA-based system implementation can depend on the availability of resources and the status quo of different settings.	**Yes** ☐<br>**Can't tell** ☑<br>**No** ☐
**10. Were all clinically important outcomes considered?**	The study examined the outcomes related to the data collection itself rather than clinical or health outcomes.	**Yes** ☐<br>**Can't tell** ☐<br>**No** ☑
**11. Are the benefits worth the harms and costs?**	Considering the efficiency of the workflow, the PDA-based system can be beneficial but the costs should be thoroughly assessed.	**Yes** ☐<br>**Can't tell** ☑<br>**No** ☐

Reference

Mohammed S, Glennerster R, Khan AJ. Impact of a Daily SMS Medication Reminder System on Tuberculosis Treatment Outcomes: A Randomized Controlled Trial. PLoS One. 2016 Nov 1;11(11):e0162944. doi: 10.1371/journal.pone.. eCollection 2016.

Section A: Are the results of the trial valid?

Question	Comments	Answer
**1. Did the trial address a clearly focused issue?**	The aim of the study was clearly stated: "To measure the impact of Zindagi SMS, a two-way SMS reminder system, on treatment success of people with drug-sensitive tuberculosis."	**Yes** ☑<br>**Can't tell** ☐<br>**No** ☐
**2. Was the assignment of patients to treatments randomized?**	The study was designed as randomized controlled trial: "Individual participants were randomized to either the Zindagi SMS or control groups, using predetermined list on the study server that was generated using simple randomization."	**Yes** ☑<br>**Can't tell** ☐<br>**No** ☐
**3. Were all of the patients who entered the trial properly accounted for at its conclusion?**	Although the study did not stop early, some patients asked "to leave the system or "died" during the trial.	**Yes** ☐<br>**Can't tell** ☐<br>**No** ☑
**4. Were patients, health workers and study personnel 'blind' to treatment?**	Yes, "The research team was blinded to the allocation sequence generated." However, the patients were informed of their randomization status according to the protocol.	**Yes** ☐<br>**Can't tell** ☐<br>**No** ☑
**5. Were the groups similar at the start of the trial?**	The study showed a table showing similarities between the two groups. "Both groups had similar baseline characteristics (Table 1)."	**Yes** ☑<br>**Can't tell** ☐<br>**No** ☐
**6. Aside from the experimental intervention, were the groups treated equally?**	Yes, "All study participants received the standard of care provided by their clinic."	**Yes** ☑<br>**Can't tell** ☐<br>**No** ☐

Section B: What are the results?

Question	Comments
**7. How large was the treatment effect?**	They found "no significant difference between the Zindagi SMS or control groups for treatment success (719 or 83% vs. 903 or 83%, respectively, p = 0.782)."
**8. How precise was the estimate of the treatment effect?**	No information available.

Section C: Will the results help locally?

Question	Comments	Answer
**9. Can the results be applied to the local population, or in your context?**	The sample from the study was "low-literate population" which may have affected the study results. The study may be applicable in similar settings.	**Yes** ☑<br>**Can't tell** ☐<br>**No** ☐
**10. Were all clinically important outcomes considered?**	Yes, clinically recorded treatment success as well as treatment outcomes and health outcomes were measured.	**Yes** ☑<br>**Can't tell** ☐<br>**No** ☐

**11. Are the benefits worth the harms and costs?**	For this study population, the SMS system was not effective in improving treatment success.	**Yes** ☐<br>**Can't tell** ☐<br>**No** ☑

Reference

Bassett IV, Coleman SM, Giddy J, Bogart LM, Chaisson CE, Ross D, et al. Sizanani: A Randomized Trial of Health System Navigators to Improve Linkage to HIV and TB Care in South Africa. Journal of acquired immune deficiency syndromes (1999). 2016 Oct 1;73(2):154-60. PMID: 27632145. doi: 10.1097/qai.0000000000001025.

Section A: Are the results of the trial valid?

Question	Comments	Answer
**1. Did the trial address a clearly focused issue?**	The study clearly stated its aim: "We evaluated the efficacy of health system navigators for improving linkage to HIV and tuberculosis (TB) care among newly diagnosed HIV-infected outpatients in Durban, South Africa."	**Yes** ☑<br>**Can't tell** ☐<br>**No** ☐
**2. Was the assignment of patients to treatments randomized?**	Yes, "After enrollment but before HIV testing, subjects were randomized to usual care or the health system navigator intervention."	**Yes** ☑<br>**Can't tell** ☐<br>**No** ☐
**3. Were all of the patients who entered the trial properly accounted for at its conclusion?**	Although the trial did not end early, some patients withdrew from the trial or died.	**Yes** ☐<br>**Can't tell** ☐<br>**No** ☑
**4. Were patients, health workers and study personnel 'blind' to treatment?**	The authors stated that the study "remains blinded to follow up rates and outcomes across arms," and the "randomization assignments were accessed by the enrolling research assistant electronically through locked randomization tables in a handheld device."	**Yes** ☑<br>**Can't tell** ☐<br>**No** ☐
**5. Were the groups similar at the start of the trial?**	The authors presented the baseline characteristics and stated that "Baseline demographic characteristics were balanced (Table 1)."	**Yes** ☑<br>**Can't tell** ☐<br>**No** ☐
**6. Aside from the experimental intervention, were the groups treated equally?**	Yes, the involvement of health system navigator is the experimental intervention and both arms were referred to a clinician: "Afterward, intervention arm participants were met by the health system navigator to establish a relationship, to identify perceived barriers to care, and to assess participants' coping strengths. They were then referred to a clinician for regular services. Usual care participants were referred directly to a clinician."	**Yes** ☑<br>**Can't tell** ☐<br>**No** ☐

Section B: What are the results?

Question	Comments	Answer
**7. How large was the treatment effect?**	They did not find the treatment effect: "we did not find an effect of time-limited health system navigation on rates of ART initiation and TB treatment completion among people newly diagnosed with HIV in Durban, South Africa."	
**8. How precise was the estimate of the treatment effect?**	No significant treatment effect was detected.	

Section C: Will the results help locally?

Question	Comments	Answer
**9. Can the results be applied to the local population, or in your context?**	This multisite trial that enrolled more than 4,000 participants visiting for HIV test within about 3 years could be conducted in only a few possible context.	**Yes** ☐<br>**Can't tell** ☐<br>**No** ☑
**10. Were all clinically important outcomes considered?**	The treatment completion was measured as primary outcome, which is clinically important. However, other outcomes such as treatment outcome or health outcome were not measured.	**Yes** ☑<br>**Can't tell** ☐<br>**No** ☐
**11. Are the benefits worth the harms and costs?**	The intervention of navigator was not effective in this study and the costs associated with the program should be evaluated.	**Yes** ☐<br>**Can't tell** ☐<br>**No** ☑

Reference Huang R, Ren G, Hu J. Bracelet- and self-directed observational therapy for control of tuberculosis: study protocol for a cluster randomized controlled trial. Trials. 2017 Jul 4;18(1):286. doi: 10.1186/s13063-017-1996-2.

Section A: Are the results of the trial valid?

Question	Comments	Answer
**1. Did the trial address a clearly focused issue?**	The authors stated the aim of the study: "We will assess whether BSDOT using a novel pillbox and smartphone application increases the adherence of poor, TB-infected, rural subjects living in mountainous regions to antibacterial drug-based treatment regimens."	**Yes** ☑<br>**Can't tell** ☐<br>**No** ☐
**2. Was the assignment of patients to treatments randomized?**	Yes, "We will conduct a cluster 1: 1 randomized trial in this area."	**Yes** ☑<br>**Can't tell** ☐<br>**No** ☐
**3. Were all of the patients who entered the trial properly accounted for at its conclusion?**	This is a study protocol, so future study can provide more information in this regard.	**Yes** ☐<br>**Can't tell** ☑<br>**No** ☐
**4. Were patients, health workers and study personnel 'blind' to treatment?**	Due to the design of the study, blinding was very limited: "Participating village physicians and patients with TB cannot be blinded; the BSDOT intervention features	**Yes** ☐<br>**Can't tell** ☐<br>**No** ☑

	open participation. We will blind the research group members involved in data analysis.”	
**5. Were the groups similar at the start of the trial?**	This is a study protocol and the recruitment did not start when the manuscript was written.	**Yes ☐<br>Can't tell ☑<br>No ☐**
**6. Aside from the experimental intervention, were the groups treated equally?**	Yes, “All participants will receive a monthly multidisciplinary check-up and will be followed up for 6 months, during which time adherence will be monitored.”	**Yes ☑<br>Can't tell ☐<br>No ☐**

Section B: What are the results?

Question	Comments	Answer
**7. How large was the treatment effect?**	This is a study protocol and therefore, no information is available yet.	
**8. How precise was the estimate of the treatment effect?**	This is a study protocol and therefore, no information is available yet.	

Section C: Will the results help locally?

Question	Comments	Answer
**9. Can the results be applied to the local population, or in your context?**	The prevalence of TB in the study setting could be different from other context.	**Yes ☐<br>Can't tell ☑<br>No ☐**
**10. Were all clinically important outcomes considered?**	Treatment outcome as well as adherence, quality of life were measured, which are clinically important outcomes for TB.	**Yes ☑<br>Can't tell ☐<br>No ☐**
**11. Are the benefits worth the harms and costs?**	The use of smartphone-based DOT can be beneficial with little possibility of direct harms to the patients. However, the implementation cost should be considered.	**Yes ☐<br>Can't tell ☑<br>No ☐**

**Reference**	Bediang G, Stoll B, Elia N, Abena JL, Geissbuhler A. SMS reminders to improve adherence and cure of tuberculosis patients in Cameroon (TB-SMS Cameroon): a randomised controlled trial. BMC Public Health. 2018 May 2;18(1):583. PMID: 29720146. doi: 10.1186/s12889-018-5502-x.

Section A: Are the results of the trial valid?

Question	Comments	Answer
**1. Did the trial address a clearly focused issue?**	The authors clearly stated the study aim: “This study aimed to evaluate the effectiveness of daily Short Message Service reminders to increase adherence and the proportion of adult tuberculosis patients cured after 6 months of treatment.”	**Yes ☑<br>Can't tell ☐<br>No ☐**

**2. Was the assignment of patients to treatments randomized?**	Yes, "This code was assigned consecutively over the recruitment of patients, who were also stratified by recruitment centres (use of random block sizes), and randomised into the two groups (intervention and control groups) with an allocation ratio of 1:1. Randomisation was carried out by research team using a computer generated list."	**Yes** ☑<br>**Can't tell** ☐<br>**No** ☐
**3. Were all of the patients who entered the trial properly accounted for at its conclusion?**	The authors mentioned that the drop-out rate was high.	**Yes** ☐<br>**Can't tell** ☐<br>**No** ☑
**4. Were patients, health workers and study personnel 'blind' to treatment?**	The study personnel and patients could not be blinded by the study design but the healthcare professionals were blinded. "Once a patient was recruited by a healthcare professional, the study identifiers and telephone numbers were communicated to the research assistant who allocated him/her in one of the two groups according to the randomisation scheme. Thus, healthcare professionals were blinded to randomization and allocation of patients into two groups during the recruitment phase, thereby preventing allocation or selection biases."	**Yes** ☐<br>**Can't tell** ☐<br>**No** ☑
**5. Were the groups similar at the start of the trial?**	There was no significant difference in overall characteristics but the disease prevalence was different between the two groups: "There is a homogeneous distribution of participants in both groups (Table 1), except for the prevalence of TB -HIV co-infection."	**Yes** ☐<br>**Can't tell** ☐<br>**No** ☑
**6. Aside from the experimental intervention, were the groups treated equally?**	Yes, "All participants received the usual care (selective DOT)...Patients in the intervention group received, in addition, free and daily SMS reminders in French…"	**Yes** ☑<br>**Can't tell** ☐<br>**No** ☐

Section B: What are the results?

Question	Comments	Answer
**7. How large was the treatment effect?**	The study concluded that there was no significant treatment effect: "Our study suggests that SMS reminders do not increase treatment success and cure proportions."	
**8. How precise was the estimate of the treatment effect?**	No significant treatment effect was found.	

Section C: Will the results help locally?

Question	Comments	Answer
**9. Can the results be applied to the local population, or in your context?**	Study context such as technology literacy or socioeconomic status should be considered to apply the study results.	**Yes** ☐<br>**Can't tell** ☑<br>**No** ☐
**10. Were all clinically important outcomes considered?**	Yes, the treatment outcome, adherence, process measure and perception were measured in detail.	**Yes** ☑<br>**Can't tell** ☐<br>**No** ☐
**11. Are the benefits worth the harms and costs?**	The implementation of SMS intervention can provide social support for TB treatment, but as the study demonstrated, the benefits as well as cost should be further evaluated.	**Yes** ☐<br>**Can't tell** ☑<br>**No** ☐

Reference

Khachadourian V, Truzyan N, Harutyunyan A, Thompson ME, Harutyunyan T, Petrosyan V. People-centered tuberculosis care versus standard directly observed therapy: study protocol for a cluster randomized controlled trial. Trials. 2015 Jun 22;16:281.(doi):10.1186/s13063-015-0802-2.

Section A: Are the results of the trial valid?

Question	Comments	Answer
**1. Did the trial address a clearly focused issue?**	The authors clearly stated the aim of the study: "The current randomized controlled trial aims to evaluate the effectiveness over usual care of an innovative multicomponent people-centered tuberculosis-care strategy in Armenia."	**Yes** ☑<br>**Can't tell** ☐<br>**No** ☐
**2. Was the assignment of patients to treatments randomized?**	The study "performed a cluster-level random assignment of drug sensitive TB patients to intervention and control arms to mitigate potential contamination of participants in the control arm."	**Yes** ☑<br>**Can't tell** ☐<br>**No** ☐
**3. Were all of the patients who entered the trial properly accounted for at its conclusion?**	This is a study protocol, so future study can provide more information in this regard.	**Yes** ☐<br>**Can't tell** ☑<br>**No** ☐
**4. Were patients, health workers and study personnel 'blind' to treatment?**	By the intervention design, the blinding was not possible for patients and health workers but the interviewers were "blinded for the assessment of secondary outcomes at follow-up."	**Yes** ☐<br>**Can't tell** ☐<br>**No** ☑
**5. Were the groups similar at the start of the trial?**	The recruitment was not completed when the protocol was published, and the authors anticipated "completion of recruitment and data collection within 12 months from the start of the field work."	**Yes** ☐<br>**Can't tell** ☑<br>**No** ☐
**6. Aside from the experimental intervention, were the groups treated equally?**	Yes, "Patients receive the required medications for one week during the weekly visits to the tuberculosis outpatient centers. Additionally, patients	**Yes** ☑<br>**Can't tell** ☐<br>**No** ☐

	receive daily Short Message Service (SMS) reminders to take their medications and daily phone calls to assure adherence and monitoring of treatment potential side effects. Control-arm patients follow the World Health Organization - recommended directly observed treatment strategy, including daily visits to tuberculosis outpatient centers for drug-intake."	

Section B: What are the results?

Question	Comments	Answer
**7. How large was the treatment effect?**	This is a study protocol and therefore, no information is available yet.	
**8. How precise was the estimate of the treatment effect?**	This is a study protocol and therefore, no information is available yet.	

Section C: Will the results help locally?

Question	Comments	Answer
**9. Can the results be applied to the local population, or in your context?**	The results can be applied if the treatment course and outpatient clinic settings are similar.	**Yes** ☐<br>**Can't tell** ☑<br>**No** ☐
**10. Were all clinically important outcomes considered?**	Clinically important outcomes such as treatment outcome as well as adherence were considered as well as psychosocial outcomes and quality of life.	**Yes** ☑<br>**Can't tell** ☐<br>**No** ☐
**11. Are the benefits worth the harms and costs?**	Sending daily SMS messages can be encouraging for TB patients without involving much harm, but the costs of system implementation should be considered.	**Yes** ☐<br>**Can't tell** ☑<br>**No** ☐

Risk of bias assessment for mixed methods study
Criteria from Atkins S, Launiala A, Kagaha A, Smith H. (2012)

**Reference**	Chaiyachati KH, Loveday M, Lorenz S, Lesh N, Larkan LM, Cinti S, et al. A pilot study of an mHealth application for healthcare workers: poor uptake despite high reported acceptability at a rural South African community-based MDR-TB treatment program. PLoS One. 2013 May 28;8(5):e64662. doi: 10.1371/journal.pone.0064662. Print 2013.

Domain	Criteria explanation	Indicative questions	Answer

**Rigor in research conduct**	**Judgement on how carefully the research is carried out; tends to be a judgement of reporting quality**	**Is the research question clearly defined?**<br><br>**Rationale for the study design discussed?**<br><br>**Is a sampling strategy well defined and justified?**<br><br>**Is the method of data collection clearly described?**	**Yes (1) ☑**<br>**No (0) ☐**<br><br>**Yes (1) ☐**<br>**No (0) ☑**<br><br>**Yes (1) ☐**<br>**No (0) ☑**<br><br>**Yes (1) ☑**<br>**No (0) ☐**<br><br>**Total: 2**
**Study context**	**A detailed description is needed to judge wider applicability of the findings; refers to transferability**	**Detailed description of the context of the study to allow assessment of applicability to other settings?**<br><br>**Discussion of limits to wider inference?**	**Yes ☑**<br>**Can't tell ☐**<br>**No ☐**
**Analysis procedure**	**An important component of rigor and reliability**	**Is the method of analysis clearly described?**	**Yes ☑**<br>**Can't tell ☐**<br>**No ☐**
**Credibility**	**Judgement on how well the findings are presented and how meaningful or believable they are**	**How credible are the findings?**<br><br>**Are the claims made supported by sufficient evidence?**	**Yes ☑**<br>**Can't tell ☐**<br>**No ☐**
**Depth, detail and richness of findings**	**An indication of the quality of the analysis which underlies credibility claims**	**e.g. "thick vs. thin description"?**<br><br>**Illumination of multiple perspectives/contribution of sample design?**<br><br>**Detection of underlying factors/influences or conceptual linkages?**<br><br>**Presentation of illuminating extracts/observations?**	**Yes ☑**<br>**Can't tell ☐**<br>**No ☐**
**Contribution to knowledge**	**Judgement on the relevance and potential utility of the findings in relation to policy, practice or theory**	**Clear discussion of how the research findings contribute to:**<br><br>**Understanding of uptake of the interventions?; theoretical**	**Yes ☑**<br>**Can't tell ☐**<br>**No ☐**

		**conceptions of uptake of interventions?**<br><br>**New areas of investigation identified?**	

Reference

Howard AA, Hirsch-Moverman Y, Frederix K, Daftary A, Saito S, Gross T, et al. The START Study to evaluate the effectiveness of a combination intervention package to enhance antiretroviral therapy uptake and retention during TB treatment among TB/HIV patients in Lesotho: rationale and design of a mixed-methods, cluster-randomized trial. Glob Health Action. 2016 Jun 27;9:31543. eCollection 2016.

Domain	Criteria explanation	Indicative questions	Answer
**Rigor in research conduct**	**Judgement on how carefully the research is carried out; tends to be a judgement of reporting quality**	**Is the research question clearly defined?**<br><br>**Rationale for the study design discussed?**<br><br>**Is a sampling strategy well defined and justified?**<br><br>**Is the method of data collection clearly described?**	**Yes (1) ☑**<br>**No (0) ☐**<br><br>**Yes (1) ☑**<br>**No (0) ☐**<br><br>**Yes (1) ☑**<br>**No (0) ☐**<br><br>**Yes (1) ☑**<br>**No (0) ☐**<br><br>**Total: 4**
**Study context**	**A detailed description is needed to judge wider applicability of the findings; refers to transferability**	**Detailed description of the context of the study to allow assessment of applicability to other settings?**<br><br>**Discussion of limits to wider inference?**	**Yes ☑**<br>**Can't tell ☐**<br>**No ☐**
**Analysis procedure**	**An important component of rigor and reliability**	**Is the method of analysis clearly described?**	**Yes ☑**<br>**Can't tell ☐**<br>**No ☐**
**Credibility**	**Judgement on how well the findings are presented and how meaningful or believable they are**	**How credible are the findings?**<br><br>**Are the claims made supported by sufficient evidence?**	**Yes ☐**<br>**Can't tell ☑**<br>**No ☐**

**Depth, detail and richness of findings**	**An indication of the quality of the analysis which underlies credibility claims**	**e.g. "thick vs. thin description"?**<br><br>**Illumination of multiple perspectives/contribution of sample design?**<br><br>**Detection of underlying factors/influences or conceptual linkages?**<br><br>**Presentation of illuminating extracts/observations?**	**Yes ☐**<br>**Can't tell ☑**<br>**No ☐**
**Contribution to knowledge**	**Judgement on the relevance and potential utility of the findings in relation to policy, practice or theory**	**Clear discussion of how the research findings contribute to:**<br><br>**Understanding of uptake of the interventions?; theoretical conceptions of uptake of interventions?**<br><br>**New areas of investigation identified?**	**Yes ☑**<br>**Can't tell ☐**<br>**No ☐**

**Reference**	Iribarren S, Beck S, Pearce PF, Chirico C, Etchevarria M, Cardinale D, et al. TextTB: A Mixed Method Pilot Study Evaluating Acceptance, Feasibility, and Exploring Initial Efficacy of a Text Messaging Intervention to Support TB Treatment Adherence. Tuberculosis research and treatment. 2013;2013:349394. PMID: 24455238. doi: 10.1155/2013/349394.

**Domain**	**Criteria explanation**	**Indicative questions**	**Answer**
**Rigor in research conduct**	**Judgement on how carefully the research is carried out; tends to be a judgement of reporting quality**	**Is the research question clearly defined?**<br><br>**Rationale for the study design discussed?**<br><br>**Is a sampling strategy well defined and justified?**<br><br>**Is the method of data collection clearly described?**	**Yes (1) ☑**<br>**No (0) ☐**<br><br>**Yes (1) ☐**<br>**No (0) ☑**<br><br>**Yes (1) ☐**<br>**No (0) ☑**<br><br>**Yes (1) ☑**<br>**No (0) ☐**

			**Total: 2**
**Study context**	**A detailed description is needed to judge wider applicability of the findings; refers to transferability**	**Detailed description of the context of the study to allow assessment of applicability to other settings?**<br><br>**Discussion of limits to wider inference?**	**Yes ☑**<br>**Can't tell ☐**<br>**No ☐**
**Analysis procedure**	**An important component of rigor and reliability**	**Is the method of analysis clearly described?**	**Yes ☑**<br>**Can't tell ☐**<br>**No ☐**
**Credibility**	**Judgement on how well the findings are presented and how meaningful or believable they are**	**How credible are the findings?**<br><br>**Are the claims made supported by sufficient evidence?**	**Yes ☑**<br>**Can't tell ☐**<br>**No ☐**
**Depth, detail and richness of findings**	**An indication of the quality of the analysis which underlies credibility claims**	**e.g. "thick vs. thin description"?**<br><br>**Illumination of multiple perspectives/contribution of sample design?**<br><br>**Detection of underlying factors/influences or conceptual linkages?**<br><br>**Presentation of illuminating extracts/observations?**	**Yes ☑**<br>**Can't tell ☐**<br>**No ☐**
**Contribution to knowledge**	**Judgement on the relevance and potential utility of the findings in relation to policy, practice or theory**	**Clear discussion of how the research findings contribute to:**<br><br>**Understanding of uptake of the interventions?; theoretical conceptions of uptake of interventions?**<br><br>**New areas of investigation identified?**	**Yes ☑**<br>**Can't tell ☐**<br>**No ☐**

**Reference**	Hirsch-Moverman Y, Burkot C, Saito S, Frederix K, Pitt B, Melaku Z, et al. Reaching the end of the line: Operational issues with implementing phone-based unannounced pill counts in resource-limited settings. PLoS One. 2017 Oct 19;12(10):e0185549. doi: 10.1371/journal.pone.. eCollection 2017.

**Domain**	**Criteria explanation**	**Indicative questions**	**Answer**
**Rigor in research conduct**	**Judgement on how carefully the research is carried out; tends to be a judgement of reporting quality**	**Is the research question clearly defined?**<br><br>**Rationale for the study design discussed?**<br><br>**Is a sampling strategy well defined and justified?**<br><br>**Is the method of data collection clearly described?**	**Yes (1) ☑**<br>**No (0) ☐**<br><br>**Yes (1) ☐**<br>**No (0) ☑**<br><br>**Yes (1) ☐**<br>**No (0) ☑**<br><br>**Yes (1) ☑**<br>**No (0) ☐**<br><br>**Total: 2**
**Study context**	**A detailed description is needed to judge wider applicability of the findings; refers to transferability**	**Detailed description of the context of the study to allow assessment of applicability to other settings?**<br><br>**Discussion of limits to wider inference?**	**Yes ☑**<br>**Can't tell ☐**<br>**No ☐**
**Analysis procedure**	**An important component of rigor and reliability**	**Is the method of analysis clearly described?**	**Yes ☐**<br>**Can't tell ☐**<br>**No ☑**
**Credibility**	**Judgement on how well the findings are presented and how meaningful or believable they are**	**How credible are the findings?**<br><br>**Are the claims made supported by sufficient evidence?**	**Yes ☑**<br>**Can't tell ☐**<br>**No ☐**
**Depth, detail and richness of findings**	**An indication of the quality of the analysis which underlies credibility claims**	**e.g. "thick vs. thin description"?**<br><br>**Illumination of multiple perspectives/contribution of sample design?**<br><br>**Detection of underlying factors/influences or conceptual linkages?**	**Yes ☑**<br>**Can't tell ☐**<br>**No ☐**

		**Presentation of illuminating extracts/observations?**	
**Contribution to knowledge**	**Judgement on the relevance and potential utility of the findings in relation to policy, practice or theory**	**Clear discussion of how the research findings contribute to:**<br><br>**Understanding of uptake of the interventions?; theoretical conceptions of uptake of interventions?**<br><br>**New areas of investigation identified?**	**Yes ☑**<br>**Can't tell ☐**<br>**No ☐**

Reference Hirsch-Moverman Y, Howard AA, Frederix K, Lebelo L, Hesseling A, Nachman S, et al. The PREVENT study to evaluate the effectiveness and acceptability of a community-based intervention to prevent childhood tuberculosis in Lesotho: study protocol for a cluster randomized controlled trial. Trials. 2017 Nov 21;18(1):552. doi: 10.1186/s13063-017-2184-0.

Domain	Criteria explanation	Indicative questions	Answer
**Rigor in research conduct**	**Judgement on how carefully the research is carried out; tends to be a judgement of reporting quality**	**Is the research question clearly defined?**<br><br>**Rationale for the study design discussed?**<br><br>**Is a sampling strategy well defined and justified?**<br><br>**Is the method of data collection clearly described?**	**Yes (1) ☑**<br>**No (0) ☐**<br><br>**Yes (1) ☑**<br>**No (0) ☐**<br><br>**Yes (1) ☑**<br>**No (0) ☐**<br><br>**Yes (1) ☑**<br>**No (0) ☐**<br><br>**Total: 4**
**Study context**	**A detailed description is needed to judge wider applicability of the findings; refers to transferability**	**Detailed description of the context of the study to allow assessment of applicability to other settings?**<br><br>**Discussion of limits to wider inference?**	**Yes ☑**<br>**Can't tell ☐**<br>**No ☐**

**Analysis procedure**	**An important component of rigor and reliability**	**Is the method of analysis clearly described?**	**Yes ☑<br>Can't tell ☐<br>No ☐**
**Credibility**	**Judgement on how well the findings are presented and how meaningful or believable they are**	**How credible are the findings?**<br><br>**Are the claims made supported by sufficient evidence?**	**Yes ☐<br>Can't tell ☑<br>No ☐**
**Depth, detail and richness of findings**	**An indication of the quality of the analysis which underlies credibility claims**	**e.g. "thick vs. thin description"?**<br><br>**Illumination of multiple perspectives/contribution of sample design?**<br><br>**Detection of underlying factors/influences or conceptual linkages?**<br><br>**Presentation of illuminating extracts/observations?**	**Yes ☐<br>Can't tell ☑<br>No ☐**
**Contribution to knowledge**	**Judgement on the relevance and potential utility of the findings in relation to policy, practice or theory**	**Clear discussion of how the research findings contribute to:**<br><br>**Understanding of uptake of the interventions?; theoretical conceptions of uptake of interventions?**<br><br>**New areas of investigation identified?**	**Yes ☐<br>Can't tell ☑<br>No ☐**

Risk of bias assessment for cohort study
CASP (Cohort Study) Checklist

Reference Nguyen TA, Pham MT, Nguyen TL, Nguyen VN, Pham DC, Nguyen BH, et al. Video Directly Observed Therapy to support adherence with treatment for tuberculosis in Vietnam: A prospective cohort study. Int J Infect Dis. 2017 Dec;65:85-9. PMID: 29030137. doi: 10.1016/j.ijid.2017.09.029.

Section A: Are the results of the study valid?

Question	Comments	Answer
**1. Did the study address a clearly focused issue?**	Yes, the study objective was clearly stated: "We aimed to evaluate the feasibility of using asynchronous Video Directly Observed Therapy (VDOT) to	**Yes ☑<br>Can't tell ☐<br>No ☐**

	support treatment adherence among patients with bacteriologically confirmed pulmonary tuberculosis.”	
**2. Was the cohort recruited in an acceptable way?**	The prospective cohort study usually involves a population without the health outcome/disease of interest and distinguish those with exposure from those without exposure. It is thus classified as an epidemiologic study rather than an interventional study, but this study recruited TB patients and involved VDOT intervention.	**Yes** ☐<br>**Can’t tell** ☐<br>**No** ☑
**3. Was the exposure accurately measured to minimize bias?**	No exposure was measured in this study.	**Yes** ☐<br>**Can’t tell** ☑<br>**No** ☐
**4. Was the outcome accurately measured to minimize bias?**	Yes, “Adherence was recorded by study staff as adequate if a participant held up all required tablets, placed them in their mouth and swallowed. Treatment adherence was assessed based upon pill count of remaining tablets. A questionnaire evaluating difficulties with using the smartphone, or the app, was completed at each visit.	**Yes** ☑<br>**Can’t tell** ☐<br>**No** ☐
**5. (a) Have the authors identified all important confounding factors?**	The authors presented baseline characteristics that can be potential confounding factors.	**Yes** ☑<br>**Can’t tell** ☐<br>**No** ☐
**5. (b) Have they taken account of the confounding factors in the design and/or analysis?**	However, the authors did not mention possibility of confounding factors.	**Yes** ☑<br>**Can’t tell** ☐<br>**No** ☐
**6. (a) Was the follow up of subjects complete enough?**	The authors stated that “two participants did not complete follow-up,” which was 5% of the total participants.	**Yes** ☑<br>**Can’t tell** ☐<br>**No** ☐
**6. (b) Was the follow up of subjects long enough?**	The follow up period was 60 days. The treatment period of tuberculosis (excluding MDR-TB cases) is usually 6 months, and therefore, it can be said that it was not enough.	**Yes** ☐<br>**Can’t tell** ☐<br>**No** ☑

Section B: What are the results?

**Question**	**Comments**	**Answer**
**7. What are the results of this study?**	“Among participating patients, 27 (71.1%) of patients took all required doses. A median of 88.4% (interquartile range 75.8%-93.7%) of doses were correctly recorded and uploaded. Participants rated the VDOT interface highly, despite facing some initial technical difficulties.”	
**8. How precise are the results?**	No information is available.	
**9. Do you believe the results?**	The authors provided descriptive results, which themselves seem credible.	**Yes** ☑<br>**Can’t tell** ☐<br>**No** ☐

Section C: Will the results help locally?

Question	Comments	Answer
**10. Can the results be applied to the local population?**	Yes, the authors described the study setting to discuss the applicability. In addition, they mentioned challenges for some populations. "familiarity with this technology may be more limited than in the general population. Older populations may also face challenges adapting to new technological tools to support adherence"	**Yes ☑ Can't tell ☐ No ☐**
**11. Do the results of this study fit with other available evidence?**	Yes, according to the study results, the VDOT has potential but there are some issues such as privacy concerns or technological difficulties.	**Yes ☑ Can't tell ☐ No ☐**
**12. What are the implications of this study for practice?**	Implementing VDOT can be effective in improving medication adherence but technological challenges and population characteristics as well as confidentiality should be considered for scale-up.	**Yes ☑ Can't tell ☐ No ☐**

Risk of bias assessment for qualitative study
CASP (Qualitative Study) Checklist

**Reference**	Daftary A, Hirsch-Moverman Y, Kassie GM, Melaku Z, Gadisa T, Saito S, et al. A Qualitative Evaluation of the Acceptability of an Interactive Voice Response System to Enhance Adherence to Isoniazid Preventive Therapy Among People Living with HIV in Ethiopia. AIDS Behav. 2017 Nov;21(11):3057-67. doi: 10.1007/s10461-016-1432-8.

Section A: Are the results of the study valid?

Question	Comments	Answer
**1. Was there a clear statement of the aims of the research?**	Yes, the authors stated the aim of this study, "This paper describes a qualitative evaluation of patient acceptability toward IVR to inform its implementation in our study setting."	**Yes ☑ Can't tell ☐ No ☐**
**2. Is a qualitative methodology appropriate?**	Yes, this study aimed to explore patient experiences and therefore, qualitative design was appropriate.	**Yes ☑ Can't tell ☐ No ☐**
**3. Was the research design appropriate to address the aims of the research?**	The authors conducted qualitative interviews with 30 participants, which was an appropriate design for the aim of the research.	**Yes ☑ Can't tell ☐ No ☐**
**4. Was the recruitment strategy appropriate to the aims of the research?**	They recruited 30 patient participants of a randomized trial, using heterogeneous sampling to recruit a diverse sample of patient participants. This was an appropriate strategy to analyze different perspectives.	**Yes ☑ Can't tell ☐ No ☐**

**5. Was the data collected in a way that addressed the research issue?**	Yes, the qualitative questions were "open-ended, exploratory questions comprised within a semi-structured interview guide , and asked in casual, non-judgmental, and culturally sensitive ways to facilitate capture of participants' perceptions and attitudes toward IVR, and perceived benefits and challenges."	**Yes ☑<br>Can't tell ☐<br>No ☐**
**6. Has the relationship between researcher and participants been adequately considered?**	Participants were informed of important information from the interviewers, including adequate opportunity to ask questions about the study.	**Yes ☑<br>Can't tell ☐<br>No ☐**

Section B: What are the results?

Question	Comments	Answer
**7. Have ethical issues been taken into consideration?**	Yes, the authors declared that the study was conducted in accordance with the ethical standards of the institutional research committee and the 1964 Helsinki declaration. Informed consent form was obtained from all participants.	**Yes ☑<br>Can't tell ☐<br>No ☐**
**8. Was the data analysis sufficiently rigorous?**	Yes, the recordings "were transcribed verbatim, translated, anonymized, and thematically analyzed using a grounded theory framework."	**Yes ☑<br>Can't tell ☐<br>No ☐**
**9. Is there a clear statement of findings?**	Yes, "four themes emerged from our data: satisfaction with automated calls, maintaining HIV confidentiality, preferences for calls versus visits, and literacy related to IVR technology."	**Yes ☑<br>Can't tell ☐<br>No ☐**

Section C: Will the results help locally?

Question	Comments
**10. How valuable is the research?**	The research was rigorously conducted and provided valuable evidence on patient experience for IVR (interactive voice response) to improve medication adherence.

**Reference**	Albino S, Tabb KM, Requena D, Egoavil M, Pineros-Leano MF, Zunt JR, et al. Perceptions and acceptability of short message services technology to improve treatment adherence amongst tuberculosis patients in Peru: a Focus Group Study. PLoS One. 2014 May 14;9(5):e95770. doi: 10.1371/journal.pone.0095770. eCollection 2014.

Section A: Are the results of the study valid?

Question	Comments	Answer
**1. Was there a clear statement of the aims of the research?**	The authors clearly stated the aim of the study, "we sought to investigate perceptions related to feasibility and acceptability of using text messaging to	**Yes ☑<br>Can't tell ☐<br>No ☐**

	improve treatment adherence among adults who were receiving treatment for TB in Callao, Peru.”	
**2. Is a qualitative methodology appropriate?**	Yes, the study aimed to “understand the attitudes, perceptions, and feasibility of using short message service (SMS) reminders to improve TB treatment adherence,” which could be investigated well with a qualitative design.	**Yes ☑**<br>**Can’t tell ☐**<br>**No ☐**
**3. Was the research design appropriate to address the aims of the research?**	Yes, they conducted “focus group qualitative interviews with current TB positive and non-contagious participants” which was an appropriate approach for the study aim.	**Yes ☑**<br>**Can’t tell ☐**<br>**No ☐**
**4. Was the recruitment strategy appropriate to the aims of the research?**	Yes, they “recruited a convenience sample of TB patients currently in treatment at health clinics in the region of Callao who had completed at least 2 weeks of treatment prior to consenting to the study.”	**Yes ☑**<br>**Can’t tell ☐**<br>**No ☐**
**5. Was the data collected in a way that addressed the research issue?**	Yes, they “conducted four focus groups with TB patients” using a “a semi-structured interview guide” and collected socio-demographic data as well. “Focus group interviews lasted an average of 50 minutes.”	**Yes ☑**<br>**Can’t tell ☐**<br>**No ☐**
**6. Has the relationship between researcher and participants been adequately considered?**	Yes, the recruiting nurses were trained intensively and the participation was voluntary with no compensation involved. A trained facilitator and secondary facilitator (note taker) were led the interview.	**Yes ☑**<br>**Can’t tell ☐**<br>**No ☐**

Section B: What are the results?

Question	Comments	Answer
**7. Have ethical issues been taken into consideration?**	Yes, the study was approved by the institutional review board, and the verbal informed consent was obtained to avoid issues with literacy. Also, “personal information such as names or other identifiers was not recorded.”	**Yes ☑**<br>**Can’t tell ☐**<br>**No ☐**
**8. Was the data analysis sufficiently rigorous?**	Yes, they “used thematic network analysis and a codebook technique to conduct qualitative analysis of the transcripts for four focus groups. Three raters (raters include the following authors S.A.,K.T., D.R.) read the transcripts and developed a coding framework.”	**Yes ☑**<br>**Can’t tell ☐**<br>**No ☐**
**9. Is there a clear statement of findings?**	Yes, “Three major themes emerged from the data: limits on health literacy and information posed challenges to	**Yes ☑**<br>**Can’t tell ☐**

	successful TB treatment adherence, treatment motivation at times facilitated adherence to TB treatment, and acceptability of SMS including positive perceptions of SMS to improve TB treatment adherence.”	**No** ☐

Section C: Will the results help locally?

Question	Comments
**10. How valuable is the research?**	The research findings can inform SMS technology for TB treatment adherence in other low-resource settings.

**Reference**	Nhavoto JA, Gronlund A, Klein GO. Mobile health treatment support intervention for HIV and tuberculosis in Mozambique: Perspectives of patients and healthcare workers. PLoS One. 2017 Apr 18;12(4):e0176051. doi: 10.1371/journal.pone.. eCollection 2017.

Section A: Are the results of the study valid?

Question	Comments	Answer
**1. Was there a clear statement of the aims of the research?**	Yes, “this study investigates perspectives of patients and HCWs regarding SMS use in a randomised control trial (RCT) aiming at improving patient retention in HIV and TB-HIV care. We also investigate if and how demographics affect patients' attitudes towards the SMS communication.”	**Yes** ☑<br>**Can’t tell** ☐<br>**No** ☐
**2. Is a qualitative methodology appropriate?**	Yes, the study aimed to examine different perspectives by using a qualitative study design.	**Yes** ☑<br>**Can’t tell** ☐<br>**No** ☐
**3. Was the research design appropriate to address the aims of the research?**	Yes, “a total of 141 patients and 40 HCWs were interviewed” to provide sufficient evidence for analyzing different perspectives.	**Yes** ☑<br>**Can’t tell** ☐<br>**No** ☐
**4. Was the recruitment strategy appropriate to the aims of the research?**	The study involved five recruitment sites that provide ART and TB care within 2 RCTs, so there could be systematic heterogeneity among study populations. In fact, the authors mentioned it as their limitation.	**Yes** ☐<br>**Can’t tell** ☐<br>**No** ☑
**5. Was the data collected in a way that addressed the research issue?**	Yes, “the interview guide for the patients and the questionnaire for the HCW were developed and pilot tested before data collection.” The “respondents rated usefulness, perceived benefits, ease of use, satisfaction, and risks of the SMS system using a Likert scale questionnaire. A semi-structured interview guide was followed.”	**Yes** ☑<br>**Can’t tell** ☐<br>**No** ☐

**6. Has the relationship between researcher and participants been adequately considered?**	Limited information was provided regarding the relationship between research and participants.	**Yes** ☐<br>**Can't tell** ☑<br>**No** ☐

Section B: What are the results?

Question	Comments	Answer
**7. Have ethical issues been taken into consideration?**	Yes, the study was approved by the relevant ethical review boards. Also, "all participants provided written or verbal informed consent in Portuguese before their enrolment."	**Yes** ☑<br>**Can't tell** ☐<br>**No** ☐
**8. Was the data analysis sufficiently rigorous?**	Yes, the interview conducted by two health experts were transcribed. "The first author checked the transcriptions to ensure consistency with the recordings and translated the transcriptions into English. For qualitative analysis, data were coded and underwent thematic content analysis."	**Yes** ☑<br>**Can't tell** ☐<br>**No** ☐
**9. Is there a clear statement of findings?**	Yes, "Both patients and HCW found the SMS system useful and reliable. Most highly rated positive effects were reducing the number of failures to collect medication and avoiding missing appointments. Patients' confidence in the system was high."	**Yes** ☑<br>**Can't tell** ☐<br>**No** ☐

Section C: Will the results help locally?

Question	Comments
**10. How valuable is the research?**	The study provides evidence on how patients and healthcare workers perceive the SMS system for HIV and TB treatment adherence. It is valuable in that it discusses two different perspectives at the same time.

Risk of bias assessment for observational study/implementation project CASP (Case Control Study) Checklist

**Reference**	Hoffman JA, Cunningham JR, Suleh AJ, Sundsmo A, Dekker D, Vago F, et al. Mobile direct observation treatment for tuberculosis patients: a technical feasibility pilot using mobile phones in Nairobi, Kenya. American journal of preventive medicine. 2010 Jul;39(1):78-80. PMID: 20537846. doi: 10.1016/j.amepre.2010.02.018.

Section A: Are the results of the trial valid?

Question	Comments	Answer
**1. Did the study address a clearly focused issue?**	Yes, "the primary objective was to assess technical feasibility, including patient and health provider receptivity to remote DOT through mobile video. The	**Yes** ☑<br>**Can't tell** ☐<br>**No** ☐

	secondary objective was to assess patient preferences and receptivity to receiving TB health messages on a mobile phone.”	
**2. Did the authors use an appropriate method to answer their question?**	Yes, the authors claimed that the study is a “a proof-of-concept pilot designed to provide remote Mobile Direct Observation of Treatment (MDOT) for TB patients,” and they designed pilot MDOT program with 13 patients.	**Yes ☑**<br>**Can’t tell ☐**<br>**No ☐**
**3. Were the cases recruited in an acceptable way?**	Yes, they used convenient sampling for it is a pilot study - “three healthcare professionals along with 13 patients and their treatment supporters were recruited from the Mbagathi District Hospital in Nairobi, Kenya.”	**Yes ☑**<br>**Can’t tell ☐**<br>**No ☐**
**4. Were the controls selected in an acceptable way?**	Not applicable.	**Yes ☐**<br>**Can’t tell ☑**<br>**No ☐**
**5. Was the exposure accurately measured to minimize bias?**	Since this is a proof-of-concept study, they did not take into account the potential bias.	**Yes ☐**<br>**Can’t tell ☐**<br>**No ☑**
**6. (a) Aside from the experimental intervention, were the groups treated equally?**	Not applicable.	
**6. (b) Have the authors taken account of the potential confounding factors in the design and/or in their analysis?**	Since this is a proof-of-concept study, they did not take into account the potential confounding factors.	**Yes ☐**<br>**Can’t tell ☐**<br>**No ☑**

Section B: What are the results?

Question	Comments	Answer
**7. How large was the treatment effect?**	“All three health professionals and 11 patients completed the trial. All agreed that MDOT was a viable option, and eight patients preferred MDOT to clinic DOT”	
**8. How precise was the estimate of the treatment effect?**	They presented overall average without the actual numbers or tables, which may prevent from evaluating accuracy of the data.	
**9. Do you believe the results?**	The descriptive analysis presented some negative results as well as positive aspect of the intervention.	**Yes ☑**<br>**No ☐**

Section C: Will the results help locally?

Question	Comments	Answer
**10. Can the results be applied to the local population?**	No, the authors claimed that their results “are not generalizable.”	**Yes ☐**<br>**Can’t tell ☐**<br>**No ☑**

**11. Do the results of this study fit with other available evidence?**	Yes, the general satisfaction with the system itself has been reported in other studies as well.	**Yes** ☑<br>**Can't tell** ☐<br>**No** ☐

**Reference**	de Sumari-de Boer IM, van den Boogaard J, Ngowi KM, Semvua HH, Kiwango KW, Aarnoutse RE, et al. Feasibility of Real Time Medication Monitoring Among HIV Infected and TB Patients in a Resource-Limited Setting. AIDS Behav. 2016 May;20(5):1097-107. doi: 10.07/s10461-015-1254-0.

Section A: Are the results of the trial valid?

Question	Comments	Answer
**1. Did the study address a clearly focused issue?**	Yes, it is a "a pilot-study on real time medication monitoring (RTMM) in a resource-limited setting."	**Yes** ☑<br>**Can't tell** ☐<br>**No** ☐
**2. Did the authors use an appropriate method to answer their question?**	They used "a prospective single-arm observational pilot study" design involving both quantitative data and qualitative analysis to determine feasibility."<br>It is unclear whether this was a mixed method study or a pilot observational study.	**Yes** ☐<br>**Can't tell** ☐<br>**No** ☑
**3. Were the cases recruited in an acceptable way?**	They "recruited five treatment experienced HIV infected patients in the Infectious Diseases Clinic (IDC) of Kilimanjaro Christian Medical Center (KCMC, Moshi, Tanzania) and five treatment experienced TB patients in the TB clinic of Mawenzi hospital in Moshi, Tanzania." Since this is a pilot study, detailed information on recruitment was not provided and the sample size is relatively small.	**Yes** ☐<br>**Can't tell** ☐<br>**No** ☑
**4. Were the controls selected in an acceptable way?**	Not applicable.	**Yes** ☐<br>**Can't tell** ☑<br>**No** ☐
**5. Was the exposure accurately measured to minimize bias?**	Since this is a feasibility study, they did not take into account the potential bias.	**Yes** ☐<br>**Can't tell** ☐<br>**No** ☑
**6. (a) Aside from the experimental intervention, were the groups treated equally?**	Not applicable.	
**6. (b) Have the authors taken account of the potential confounding factors in the design and/or in their analysis?**	Since this is a feasibility study, they did not take into account the potential confounding factors.	**Yes** ☐<br>**Can't tell** ☐<br>**No** ☑

Section B: What are the results?

Question	Comments	Answer
**7. How large was the treatment effect?**	"Six patients (60 %) reached adherence of[95 %. Nine-hundred-twenty-two of 1104 intakes (84 %) were on time. Five-hundred reminders (45 %) were sent, of which 202 (40 %) were incorrect, because of an unstable mobile network. Nine patients found the device helpful and nine mentioned it keeps medication safe. Six patients reported that the size was too big."	
**8. How precise was the estimate of the treatment effect?**	Important variables were measured such as "percentage of doses taken on time, percentage of sent reminders (divided by total intake prescription), percentage of correct reminders (after missed doses), or percentage of incorrect reminders." However, the result table for the quantitative data were not presented.	
**9. Do you believe the results?**	The results from this feasibility study should be further investigated with more rigorous design and larger sample.	**Yes** ☐<br>**No** ☑

Section C: Will the results help locally?

Question	Comments	Answer
**10. Can the results be applied to the local population?**	As the authors discussed, mobile phone ownership and other socioeconomic status may affect the feasibility of the study in other resource-limited settings.	**Yes** ☐<br>**Can't tell** ☐<br>**No** ☑
**11. Do the results of this study fit with other available evidence?**	Yes, this pilot study concluded that real-time medication monitoring is feasible and acceptable, which were suggested by other studies as well.	**Yes** ☑<br>**Can't tell** ☐<br>**No** ☐

Reference Garfein RS, Collins K, Munoz F, Moser K, Cerecer-Callu P, Raab F, et al. Feasibility of tuberculosis treatment monitoring by video directly observed therapy: a binational pilot study. Int J Tuberc Lung Dis. 2015 Sep;19(9):1057-64. doi: 10.5588/ijtld.14.0923.

Section A: Are the results of the trial valid?

Question	Comments	Answer
**1. Did the study address a clearly focused issue?**	Yes, the authors stated that they "evaluated the feasibility and acceptability of "Video DOT" (VDOT), which allowed patients to record and transmit medication ingestion videos that were watched remotely by healthcare providers to document adherence."	**Yes** ☑<br>**Can't tell** ☐<br>**No** ☐
**2. Did the authors use an appropriate method to answer their question?**	Yes, they "conducted a single-arm trial among TB patients in San Diego, CA (n=43) and Tijuana, B.C., Mexico (n=9) to represent high- and low-resources settings and the pre/post treatment interviews were also conducted." The	**Yes** ☑<br>**Can't tell** ☐<br>**No** ☐

	feasibility and acceptability can be assessed using this pilot design.	
**3. Were the cases recruited in an acceptable way?**	As for the feasibility study design, the recruitment was acceptable. "TB Control Program staff in both cities recruited individuals currently receiving treatment for confirmed or suspected pulmonary TB. Patients who met the eligibility criteria were sequentially enrolled."	**Yes ☑**<br>**Can't tell ☐**<br>**No ☐**
**4. Were the controls selected in an acceptable way?**	Not applicable.	**Yes ☐**<br>**Can't tell ☑**<br>**No ☐**
**5. Was the exposure accurately measured to minimize bias?**	Although the authors discussed the measures to minimize response bias in the interviews, they did not mention bias regarding exposure.	**Yes ☐**<br>**Can't tell ☑**<br>**No ☐**
**6. (a) Aside from the experimental intervention, were the groups treated equally?**	Not applicable.	
**6. (b) Have the authors taken account of the potential confounding factors in the design and/or in their analysis?**	Since this is a feasibility study, the potential of confounding factors was not discussed.	**Yes ☐**<br>**Can't tell ☐**<br>**No ☑**

Section B: What are the results?

Question	Comments	Answer
**7. How large was the treatment effect?**	"Mean adherence was 93% (51%–100%) in San Diego and 96% (88%–100%) in Tijuana. Overall, 92% reported never/rarely having problems recording videos, 92% preferred VDOT over in-person DOT, 84% thought VDOT was more confidential and 100% said they would recommend VDOT to others."	
**8. How precise was the estimate of the treatment effect?**	Not enough information was provided to assess the accuracy of the estimate.	
**9. Do you believe the results?**	As the authors stated that "patient selection could have been biased toward adherent patients limiting generalizability of adherence rates to all TB patients; however, such bias is unlikely to have affected satisfaction with VDOT." However, this could have influenced the study results.	**Yes ☐**<br>**No ☑**

Section C: Will the results help locally?

Question	Comments	Answer

**10. Can the results be applied to the local population?**	Further studies should be conducted to evaluate the generalizability of the VDOT.	**Yes ☐**<br>**Can't tell ☑**<br>**No ☐**
**11. Do the results of this study fit with other available evidence?**	The authors concluded that VDOT is feasible and their study results may be used in other resource-limited settings where "in-person DOT is impractical." This conclusion is in line with other feasibility studies.	**Yes ☑**<br>**Can't tell ☐**<br>**No ☐**

Reference Dwolatzky B, Trengove E, Struthers H, McIntyre JA, Martinson NA. Linking the global positioning system (GPS) to a personal digital assistant (PDA) to support tuberculosis control in South Africa: a pilot study. International journal of health geographics. 2006 Aug 16;5:34. PMID: 16911806. doi: 10.1186/1476-072x-5-34.

Section A: Are the results of the trial valid?

Question	Comments	Answer
**1. Did the study address a clearly focused issue?**	Yes, this study "assessed the feasibility of using a handheld computing device programmed with customised software and linked to a GPS receiver, to assist TB control programmes to trace patients who interrupt treatment in areas without useful street maps."	**Yes ☑**<br>**Can't tell ☐**<br>**No ☐**
**2. Did the authors use an appropriate method to answer their question?**	They conducted a "proof of concept study to compare the time taken to re-find a home comparing given residential addresses with a customised personalised digital assistant." As a proof of concept study, the feasibility was assessed.	**Yes ☑**<br>**Can't tell ☐**<br>**No ☐**
**3. Were the cases recruited in an acceptable way?**	The participants were recruited from two clinics but no further information was given regarding recruitment.	**Yes ☐**<br>**Can't tell ☑**<br>**No ☐**
**4. Were the controls selected in an acceptable way?**	Not applicable.	**Yes ☐**<br>**Can't tell ☑**<br>**No ☐**
**5. Was the exposure accurately measured to minimize bias?**	As the authors discussed, there could be bias among research assistants compared to community health workers in terms of prior knowledge on the community.	**Yes ☐**<br>**Can't tell ☐**<br>**No ☑**
**6. (a) Aside from the experimental intervention, were the groups treated equally?**	Not applicable.	
**6. (b) Have the authors taken account of the potential confounding factors in the design and/or in their analysis?**	Since this is a proof of concept study, the authors did not take into account the confounding factors.	**Yes ☐**<br>**Can't tell ☐**<br>**No ☑**

Section B: What are the results?

Question	Comments	Answer
**7. How large was the treatment effect?**	Time taken to locate the ten households was reduced by 20% and 50% in each community respectively using the PDA/GPS device.	
**8. How precise was the estimate of the treatment effect?**	The authors did not provide detailed information on the time taken to locate the houses and therefore the accuracy of the estimate cannot be evaluated.	
**9. Do you believe the results?**	Since the research assistant unfamiliar with the community may have had difficulty in finding home compared to community health workers, the results may have been biased.	**Yes** ☐<br>**No** ☑

Section C: Will the results help locally?

Question	Comments	Answer
**10. Can the results be applied to the local population?**	The evidence from the study is preliminary and further studies on the feasibility should be conducted.	**Yes** ☐<br>**Can't tell** ☑<br>**No** ☐
**11. Do the results of this study fit with other available evidence?**	Only a few similar studies have been conducted so far and therefore, it is difficult to determine whether the results fit with other evidence.	**Yes** ☐<br>**Can't tell** ☑<br>**No** ☐

Reference Ha YP, Tesfalul MA, Littman-Quinn R, Antwi C, Green RS, Mapila TO, et al. Evaluation of a Mobile Health Approach to Tuberculosis Contact Tracing in Botswana. J Health Commun. 2016 Oct;21(10):1115-21. doi: 10.080/10810730.2016.1222035. Epub 2016 Sep 26.

Section A: Are the results of the trial valid?

Question	Comments	Answer
**1. Did the study address a clearly focused issue?**	Yes, the study aimed to "to develop and evaluate an mHealth approach that addresses many of the limitations of the paper form–based approach used in Botswana."	**Yes** ☑<br>**Can't tell** ☐<br>**No** ☐
**2. Did the authors use an appropriate method to answer their question?**	The study "identified and addressed operational considerations for implementation" as part of an implementation project.	**Yes** ☑<br>**Can't tell** ☐<br>**No** ☐
**3. Were the cases recruited in an acceptable way?**	The authors recruited patients from a practical perspective. "For both approaches, the same TB contact tracing team composed of two male health care workers operated as a unit and made home visits to all adult TB cases diagnosed at one of six health care facilities"	**Yes** ☑<br>**Can't tell** ☐<br>**No** ☐

**4. Were the controls selected in an acceptable way?**	Not applicable.	**Yes** ☐<br>**Can't tell** ☑<br>**No** ☐
**5. Was the exposure accurately measured to minimize bias?**	Issues regarding potential bias was not discussed.	**Yes** ☐<br>**Can't tell** ☐<br>**No** ☑
**6. (a) Aside from the experimental intervention, were the groups treated equally?**	Not applicable.	
**6. (b) Have the authors taken account of the potential confounding factors in the design and/or in their analysis?**	Since this is an implementation project, any confounding factors were considered in the study design.	**Yes** ☐<br>**Can't tell** ☐<br>**No** ☑

Section B: What are the results?

Question	Comments	Answer
**7. How large was the treatment effect?**	"The median time required to complete TB contact tracing was significantly greater for the paper form–based approach than for the mHealth approach: 5.0 min per contact (interquartile range [IQR]: 4.0–8.0) versus 2.8 min per contact (IQR: 1.7–4.4), respectively ($p < .001$)."	
**8. How precise was the estimate of the treatment effect?**	The estimated time saved from mHealth approach seemed precise (IQR and p-value) but the authors did not discuss the possibility of measurement error and other issues relating to the accuracy of the estimate.	
**9. Do you believe the results?**	The results showed that mHealth approach increased efficiency in the workflow, which seemed reasonable.	**Yes** ☑<br>**No** ☐

Section C: Will the results help locally?

Question	Comments	Answer
**10. Can the results be applied to the local population?**	The study can provide evidence on practical issues for mHealth implementation in other settings.	**Yes** ☑<br>**Can't tell** ☐<br>**No** ☐
**11. Do the results of this study fit with other available evidence?**	Currently, the evidence on the mHealth for TB contact tracing in low-resource settings is limited and further research should be conducted.	**Yes** ☐<br>**Can't tell** ☑<br>**No** ☐

**Reference**	Cowan J, Michel C, Manhica I, Mutaquiha C, Monivo C, Saize D, et al. Remote monitoring of Xpert(R) MTB/RIF testing in Mozambique: results of programmatic implementation of GxAlert. Int J Tuberc Lung Dis. 2016 Mar;20(3):335-41. doi: 10.5588/ijtld.15.0535.

Section A: Are the results of the trial valid?

Question	Comments	Answer
**1. Did the study address a clearly focused issue?**	Yes, the study objective was "to describe recently developed applications that allow for real-time, remote monitoring of Xpert results, and initial implementation of one of these products in central Mozambique."	**Yes ☑**<br>**Can't tell ☐**<br>**No ☐**
**2. Did the authors use an appropriate method to answer their question?**	The authors descriptively "evaluated three remote monitoring platforms for GeneXpert: Cepheid RemoteXpert, XpertSMS, and GxAlert." Since this is an implementation project, this approach was appropriate.	**Yes ☑**<br>**Can't tell ☐**<br>**No ☐**
**3. Were the cases recruited in an acceptable way?**	The study did not recruit patients but followed the clinical workflow to review the patient health information. This is feasible in an implementation project.	**Yes ☑**<br>**Can't tell ☐**<br>**No ☐**
**4. Were the controls selected in an acceptable way?**	Not applicable.	**Yes ☐**<br>**Can't tell ☑**<br>**No ☐**
**5. Was the exposure accurately measured to minimize bias?**	Since this is am implementation project, potential bias was not considered.	**Yes ☐**<br>**Can't tell ☐**<br>**No ☑**
**6. (a) Aside from the experimental intervention, were the groups treated equally?**	Not applicable.	
**6. (b) Have the authors taken account of the potential confounding factors in the design and/or in their analysis?**	Since this is am implementation project, potential confounding factors were not considered.	**Yes ☐**<br>**Can't tell ☐**<br>**No ☑**

Section B: What are the results?

Question	Comments	Answer
**7. How large was the treatment effect?**	Rather than measuring effect size, this study described the results of system implementation. "GxAlert software was successfully installed on all five Xpert computers, and test results are now uploaded daily via a USB internet modem to a secure online database. A password-protected web-based interface allows real-time analysis of test results, and 1200 positive tests for tuberculosis generated 8000 SMS result notifications to key individuals."	
**8. How precise was the estimate of the treatment effect?**	The level of precision can only be assessed in terms of the details provided in the study. The authors thoroughly compared	
**9. Do you believe the results?**	This comparative analysis of the three different tools provided detailed practical information, implying credibility.	**Yes ☑**<br>**No ☐**

Section C: Will the results help locally?

Question	Comments	Answer
**10. Can the results be applied to the local population?**	The results can inform important decisions for clinical settings of similar context.	**Yes** ☑<br>**Can't tell** ☐<br>**No** ☐
**11. Do the results of this study fit with other available evidence?**	More studies should be available regarding different systems of TB data platform.	**Yes** ☐<br>**Can't tell** ☑<br>**No** ☐

**Reference**	Kunawararak P, Pongpanich S, Chantawong S, Pokaew P, Traisathit P, Srithanaviboonchai K, et al. Tuberculosis treatment with mobile-phone medication reminders in northern Thailand. The Southeast Asian journal of tropical medicine and public health. 2011 Nov;42(6):1444-51. PMID: 22299414.

Section A: Are the results of the trial valid?

Question	Comments	Answer
**1. Did the study address a clearly focused issue?**	Yes, the study "aimed to study the effect of mobile phone reminders on the control of MDR-TB in northern Thailand."	**Yes** ☑<br>**Can't tell** ☐<br>**No** ☐
**2. Did the authors use an appropriate method to answer their question?**	The authors designed two models – standard of care and standard of care plus mobile phone calls. This interventional design without randomization is appropriate for assessing the effect of mHealth in a practical setting.	**Yes** ☑<br>**Can't tell** ☐<br>**No** ☐
**3. Were the cases recruited in an acceptable way?**	The patients were recruited at a public hospital in 7 provinces. Further information on recruitment process should be provided.	**Yes** ☐<br>**Can't tell** ☑<br>**No** ☐
**4. Were the controls selected in an acceptable way?**	The baseline characteristics were not considered and randomization was not involved in selecting control group.	**Yes** ☐<br>**Can't tell** ☐<br>**No** ☑
**5. Was the exposure accurately measured to minimize bias?**	The ownership of mobile phone may have influenced the patient behavior but the authors did not take into account the potential bias.	**Yes** ☐<br>**Can't tell** ☐<br>**No** ☑
**6. (a) Aside from the experimental intervention, were the groups treated equally?**	Yes, both groups were treated "according to WHO recommendations using DOTS," "except with a daily phone call reminder to take their medication using a mobile phone."	
**6. (b) Have the authors taken account of the potential confounding factors in the design and/or in their analysis?**	Issues on confounding factors were not discussed.	**Yes** ☐<br>**Can't tell** ☐<br>**No** ☑

Section B: What are the results?

Question	Comments	Answer

**7. How large was the treatment effect?**	"In the MDR-TB group, the sputum conversion rate was 20% (95% CI 8-45) in Model 1 and 90% (95% CI 73-98) in Model 2 (p<0.001). In the non-MDR-TB group, the sputum conversion rate was 52% (95% CI 36-70) in Model 1 and 37% (95% CI 22-56) in Model 2 although the difference was not significant (p=0.221)."	
**8. How precise was the estimate of the treatment effect?**	The authors provided both confidence intervals and p-values to provide precise estimates of the study results.	
**9. Do you believe the results?**	The authors thoroughly discussed nonsignificant effect as well as significant differences between the two groups.	**Yes** ☑<br>**No** ☐

Section C: Will the results help locally?

Question	Comments	Answer
**10. Can the results be applied to the local population?**	The results can be applied in other similar settings.	**Yes** ☑<br>**Can't tell** ☐<br>**No** ☐
**11. Do the results of this study fit with other available evidence?**	As other studies claimed, the results indicated that further evidence is needed to determine the effectiveness of mobile phone reminder.	**Yes** ☑<br>**Can't tell** ☐<br>**No** ☐

**Reference**	Lorent N, Choun K, Thai S, Kim T, Huy S, Pe R, et al. Community-based active tuberculosis case finding in poor urban settlements of Phnom Penh, Cambodia: a feasible and effective strategy. PLoS One. 2014 Mar 27;9(3):e92754. doi: 10.1371/journal.pone.0092754. eCollection 2014.

Section A: Are the results of the trial valid?

Question	Comments	Answer
**1. Did the study address a clearly focused issue?**	Yes, the study "aimed to assess the feasibility of community-based ACF (active case finding) for TB among the urban poor in Cambodia and determine its impact on case detection, treatment uptake and outcome."	**Yes** ☑<br>**Can't tell** ☐<br>**No** ☐
**2. Did the authors use an appropriate method to answer their question?**	As an implementation project, the study provided the description of the implementation process.	**Yes** ☑<br>**Can't tell** ☐<br>**No** ☐
**3. Were the cases recruited in an acceptable way?**	They "selected the communities through purposeful sampling after consultation with health managers and municipal authorities" for practical reasons.	**Yes** ☑<br>**Can't tell** ☐<br>**No** ☐
**4. Were the controls selected in an acceptable way?**	Not applicable.	**Yes** ☐<br>**Can't tell** ☑<br>**No** ☐

**5. Was the exposure accurately measured to minimize bias?**	The implementation process did not consider potential bias.	**Yes ☐**<br>**Can't tell ☐**<br>**No ☑**
**6. (a) Aside from the experimental intervention, were the groups treated equally?**	Not applicable.	
**6. (b) Have the authors taken account of the potential confounding factors in the design and/or in their analysis?**	The authors provided description of the implementation process and its outcome rather than identifying the influence of confounding factors on independent or dependent variables.	**Yes ☐**<br>**Can't tell ☐**<br>**No ☑**

Section B: What are the results?

Question	Comments	Answer
**7. How large was the treatment effect?**	"Xpert testing yielded 41% and 48% additional diagnoses among presumptive HIV-associated and multidrug-resistant TB cases, respectively. The median time from sputum collection to notification (by SMS) of the first positive (microscopy or Xpert) result was 3 days (IQR 2–6). Over 94% commenced TB treatment and 81% successfully completed it."	
**8. How precise was the estimate of the treatment effect?**	The estimates were descriptive and no further information was provided to determine the level of precision.	
**9. Do you believe the results?**	The detailed implementation process described in the study establishes credibility of the results.	**Yes ☑**<br>**No ☐**

Section C: Will the results help locally?

Question	Comments	Answer
**10. Can the results be applied to the local population?**	The implementation of ACF can be applicable to other low-resource settings for improving TB case detection.	**Yes ☑**<br>**Can't tell ☐**<br>**No ☐**
**11. Do the results of this study fit with other available evidence?**	Not many studies are currently available regarding the implementation of community-based TB case detection.	**Yes ☐**<br>**Can't tell ☑**<br>**No ☐**

**Reference**	Mahmud N, Rodriguez J, Nesbit J. A text message-based intervention to bridge the healthcare communication gap in the rural developing world. Technol Health Care. 2010;18(2):137-44. doi: 10.3233/THC-2010-0576.

Section A: Are the results of the trial valid?

Question	Comments	Answer
**1. Did the study address a clearly focused issue?**	Yes, the authors "report the results of a retrospective mobile health (mHealth) pilot at St. Gabriel's Hospital in Malawi designed to eliminate many of these trips	**Yes ☑**<br>**Can't tell ☐**<br>**No ☐**

	in favor of communication via text messages.”	
**2. Did the authors use an appropriate method to answer their question?**	The authors conducted a pilot implementation project by employing “a FrontlineSMS network at St. Gabriel’s Hospital in Namitete, Malawi in an effort to break down the physician-patient communication barrier.” Pilot approach was appropriate to understand challenges in operational process.	**Yes** ☑<br>**Can’t tell** ☐<br>**No** ☐
**3. Were the cases recruited in an acceptable way?**	Since this was a small scale pilot, they used convenient sampling for recruitment –“A group of 75 CHWs working at St. Gabriel’s Hospital, as well as the HBC nurse, were each given a recycled Motorola Pebl cell phone.”	**Yes** ☑<br>**Can’t tell** ☐<br>**No** ☐
**4. Were the controls selected in an acceptable way?**	Not applicable.	**Yes** ☐<br>**Can’t tell** ☑<br>**No** ☐
**5. Was the exposure accurately measured to minimize bias?**	As the authors discussed, the “outcomes evaluation was not designed to be rigorous in its elimination of bias”	**Yes** ☐<br>**Can’t tell** ☐<br>**No** ☑
**6. (a) Aside from the experimental intervention, were the groups treated equally?**	Not applicable.	
**6. (b) Have the authors taken account of the potential confounding factors in the design and/or in their analysis?**	Since this is a small scale pilot implementation project, confounding factors were not considered in the design or analysis.	**Yes** ☐<br>**Can’t tell** ☐<br>**No** ☑

Section B: What are the results?

Question	Comments	Answer
**7. How large was the treatment effect?**	“At the end of the pilot, the hospital saved approximately 2,048 hours of worker time, $2,750 on net ($3,000 in fuel savings minus $250 in operational costs), and doubled the capacity of the tuberculosis treatment program (up to 200 patients).”	
**8. How precise was the estimate of the treatment effect?**	The authors provided descriptive statistics without detailed information.	
**9. Do you believe the results?**	It is difficult to judge whether the estimates such as time savings without detailed data on costs, time spent on each activity, etc.	**Yes** ☐<br>**No** ☑

Section C: Will the results help locally?

Question	Comments	Answer
**10. Can the results be applied to the local population?**	The authors stated that communication via text message can be a “cost-effective	**Yes** ☐<br>**Can’t tell** ☑

	solutions to communication barriers in the setting of rural hospitals in the developing world." However, more rigorous evaluation on cost-effectiveness would be needed for different contexts.	**No** ☐
**11. Do the results of this study fit with other available evidence?**	More evidence on the SMS communication between physician and patient for TB should be available to determine whether this study fits with results from other studies.	**Yes** ☐<br>**Can't tell** ☑<br>**No** ☐

**Reference**	Narasimhan P, Bakshi A, Kittusami S, Prashant S, Mathai D, Bakshi K, et al. A customized m-Health system for improving Tuberculosis treatment adherence and follow-up in south India. Health and Technology. 2014 2014/05/01;4(1):1-10. doi: 10.1007/s12553-013-0067-2.

Section A: Are the results of the trial valid?

Question	Comments	Answer
**1. Did the study address a clearly focused issue?**	Yes, the study "illustrates the delivery of a mHealth service with insights into the feasibility and applicability of deploying a voice call based reminder system for drug adherence in a resource limited setting."	**Yes** ☑<br>**Can't tell** ☐<br>**No** ☐
**2. Did the authors use an appropriate method to answer their question?**	Yes, they designed an implementation project "to facilitate effective implementation of the national TB program through a mobile phone based intervention."	**Yes** ☑<br>**Can't tell** ☐<br>**No** ☐
**3. Were the cases recruited in an acceptable way?**	As an implementation project, they recruited TB patients at the local DOTS centers.	**Yes** ☑<br>**Can't tell** ☐<br>**No** ☐
**4. Were the controls selected in an acceptable way?**	Not applicable.	**Yes** ☐<br>**Can't tell** ☑<br>**No** ☐
**5. Was the exposure accurately measured to minimize bias?**	Since this is an implementation project, the possibility of bias was not considered.	**Yes** ☐<br>**Can't tell** ☐<br>**No** ☑
**6. (a) Aside from the experimental intervention, were the groups treated equally?**	Not applicable.	
**6. (b) Have the authors taken account of the potential confounding factors in the design and/or in their analysis?**	Since this is an implementation project for designing a feasible mHealth DOTS strategy, confounding factors were not discussed.	**Yes** ☐<br>**Can't tell** ☐<br>**No** ☑

Section B: What are the results?

Question	Comments	Answer
**7. How large was the treatment effect?**	"Of the followed-up patients, 88 % have completed their full course of treatment, treatment outcome recorded as success for 84 % (includes completed and cured), 5 % of the patients have been identified as default i.e. patients who stopped taking medication midway for a consecutive period of 2 months due to medical, societal or family reasons; while 7 % were notified as deceased."	
**8. How precise was the estimate of the treatment effect?**	The level of precision cannot be evaluated without detailed information on the estimates.	
**9. Do you believe the results?**	With the detailed implementation phases described in the study, the results seem credible.	**Yes** ☑<br>**No** ☐

Section C: Will the results help locally?

Question	Comments	Answer
**10. Can the results be applied to the local population?**	Since the study demonstrated the feasibility of mHealth intervention for TB drug adherence, it can be tested in other settings.	**Yes** ☑<br>**Can't tell** ☐<br>**No** ☐
**11. Do the results of this study fit with other available evidence?**	The feasibility of mHealth for TB drug adherence has been discussed in other studies as well. However, its effectiveness and cost-effectiveness should be further studied.	**Yes** ☑<br>**Can't tell** ☐<br>**No** ☐

Risk of bias assessment for economic evaluation study
CASP (Economic Evaluation) Checklist

Reference

Broomhead S, Mars M. Retrospective return on investment analysis of an electronic treatment adherence device piloted in the Northern Cape Province. Telemed J E Health. 2012 Jan-Feb;18(1):24-31. PMID: 22150713. doi: 10.1089/tmj.2011.0143.

Section A: Is the economic evaluation valid?

Question	Comments	Answer
**1. Was a well-defined question posed?**	Yes, the authors stated that this "retrospective analysis compares the costs and health outcomes of the DOTS-SIMpill cohort with DOTS-only controls."	**Yes** ☑<br>**Can't tell** ☐<br>**No** ☐
**2. Was a comprehensive description of the competing alternatives given?**	Yes, the authors compared conventional DOTS-only strategy with DOTS plus SIMpill system, which is "a cellular telephone SMS-based medical adherence support (MAS) system developed in South Africa"	**Yes** ☑<br>**Can't tell** ☐<br>**No** ☐
**3. Does the paper provide evidence that the program**	Yes, the DOTS-SIMpill group showed improved treatment outcomes in terms of	**Yes** ☑<br>**Can't tell** ☐

**would be effective? (i.e. would the program do more good than harm?)**	smear conversion rate (62.5% vs. 38.4%, p-value=0.0403) and cure rate (75% vs. 32.3%, p-value=0.0003).	**No ☐**
**4. Were the effects of the intervention identified, measured and valued appropriately?**	The effects were measured in natural units (i.e. smear conversion rate and cure rate). This approach was appropriate because the study is a cost minimization study where "the outcomes for each intervention are equivalent"	**Yes ☑**<br>**Can't tell ☐**<br>**No ☐**

Section B: How were consequences and costs assessed and compared?

Question	Comments	Answer
**5. Were all important and relevant resources required, and health outcome costs for each alternative identified, measured in appropriate units and valued credibly?**	The costs were summarized for each alternative but the opportunity costs were not explicitly considered. They used South African Rand and converted it into USD but they did not specify which year's value was used.	**Yes ☐**<br>**Can't tell ☐**<br>**No ☑**
**6. Were costs and consequences adjusted for different times at which they occurred (discounting)?**	No, the authors claimed that "discount rates are not discussed and time delay between the pilot and the analysis may reduce the accuracy of some cost estimations.	**Yes ☐**<br>**Can't tell ☐**<br>**No ☑**
**7. What were the results of the evaluation?**	"Discounted NPV (net present value) for the hypothetical implementation starting in 2005 was ZAR 3,255,256 (US$ 493,221) while starting in 2010 resulted in a discounted NPV of ZAR 4,747,636 (US$ 487,339). This is an ROI of 23% over the 5-year period."	
**8. Was an incremental analysis of the consequences and cost of alternatives performed?**	No incremental analysis results were presented.	**Yes ☐**<br>**Can't tell ☐**<br>**No ☑**
**9. Was an adequate sensitivity analysis performed?**	No sensitivity analysis was performed.	**Yes ☐**<br>**Can't tell ☐**<br>**No ☑**

Section C: Will the results help in purchasing for local people?

Question	Comments	Answer
**10. Is the program likely to be equally effective in your context or setting?**	From this cost minimization study, it is difficult to judge whether the SIMpill system is cost-effective in other low-resource settings or not without further evidence supported by cost-effective analysis or cost-benefit analysis.	**Yes ☐**<br>**Can't tell ☑**<br>**No ☐**
**11. Are the costs translatable to your setting?**	No, costs such as lab tests, medication, device and personnel should be reevaluated to translate to other settings.	**Yes ☐**<br>**Can't tell ☐**<br>**No ☑**
**12. Is it worth doing in your setting?**	The results from this study suggests that the addition of medical adherence support using mobile devices can be an	**Yes ☑**<br>**Can't tell ☐**<br>**No ☐**

	effective tool and therefore, further evidence on cost-effectiveness should be evaluated in other settings.	

Reference Hunchangsith P, Barendregt JJ, Vos T, Bertram M. Cost-effectiveness of various tuberculosis control strategies in Thailand. Value in health : the journal of the International Society for Pharmacoeconomics and Outcomes Research. 2012 Jan-Feb;15(1 Suppl):S50-5. PMID: 22265067. doi: 10.1016/j.jval.2011.11.006.

Section A: Is the economic evaluation valid?

Question	Comments	Answer
**1. Was a well-defined question posed?**	Yes, the objective of the study was "to evaluate the cost-effectiveness of different tuberculosis control strategies in Thailand."	**Yes** ☑<br>**Can't tell** ☐<br>**No** ☐
**2. Was a comprehensive description of the competing alternatives given?**	Yes, "different tuberculosis control strategies, which included health-worker, community-member, and family-member directly observed treatment (DOT) and a mobile phone "contact-reminder" system, were compared with self-administered treatment (SAT)."	**Yes** ☑<br>**Can't tell** ☐<br>**No** ☐
**3. Does the paper provide evidence that the program would be effective? (i.e. would the program do more good than harm?)**	"Cost-effectiveness results did not clearly indicate a preference for any of the interventions analyzed." And the mobile phone intervention led to less health gain than SAT.	**Yes** ☐<br>**Can't tell** ☐<br>**No** ☑
**4. Were the effects of the intervention identified, measured and valued appropriately?**	Yes, they explained rationale and the two steps for calculating "the DALYs for each intervention."	**Yes** ☑<br>**Can't tell** ☐<br>**No** ☐

Section B: How were consequences and costs assessed and compared?

Question	Comments	Answer
**5. Were all important and relevant resources required, and health outcome costs for each alternative identified, measured in appropriate units and valued credibly?**	Yes, the authors provided detailed information on cost estimation – they used "a standardized ingredients approach, requiring information on the quantities of all resources used and their unit costs."	**Yes** ☑<br>**Can't tell** ☐<br>**No** ☐
**6. Were costs and consequences adjusted for different times at which they occurred (discounting)?**	Yes, "Health outcomes were referenced to 2005 and discounted at 3% per annum. Future costs were discounted to 2005 values by using a 3% discount rate."	**Yes** ☑<br>**Can't tell** ☐<br>**No** ☐
**7. What were the results of the evaluation?**	"Cost-effectiveness results indicate no preference for any strategy."	
**8. Was an incremental analysis of the consequences**	Yes, "an incremental cost-effectiveness ratio (ICER) was evaluated for	**Yes** ☑<br>**Can't tell** ☐

**and cost of alternatives performed?**	each intervention.”	**No ☐**
**9. Was an adequate sensitivity analysis performed?**	Yes, “ninety-five percent uncertainty intervals were determined for all outcome measures by using Monte Carlo simulation with 2000 iterations”	**Yes ☑**<br>**Can’t tell ☐**<br>**No ☐**

Section C: Will the results help in purchasing for local people?

**Question**	**Comments**	**Answer**
**10. Is the program likely to be equally effective in your context or setting?**	According to the authors’ conclusion, which DOT strategy is most cost-effective cannot be determined due to high uncertainty. This key message can inform decisions for other low-resource settings.	**Yes ☑**<br>**Can’t tell ☐**<br>**No ☐**
**11. Are the costs translatable to your setting?**	No, the “intervention costs and medical costs” should be reevaluated before translating to other settings.	**Yes ☐**<br>**Can’t tell ☐**<br>**No ☑**
**12. Is it worth doing in your setting?**	Yes, estimating cost-effectiveness of different DOT strategies in other settings may lead to different conclusions and policy decision from this study.	**Yes ☑**<br>**Can’t tell ☐**<br>**No ☐**
